# Prediction models of persistent taxane-induced peripheral neuropathy among breast cancer survivors using whole-exome sequencing

**DOI:** 10.1038/s41698-024-00594-x

**Published:** 2024-05-16

**Authors:** Kristina Engvall, Hanna Uvdal, Niclas Björn, Elisabeth Åvall-Lundqvist, Henrik Gréen

**Affiliations:** 1 Department of Oncology, Jönköping, Region Jönköping County, and Department of Biomedical and Clinical Sciences, Linköping University, Linköping, Sweden; 2 Division of Clinical Chemistry and Pharmacology, Department of Biomedical and Clinical Sciences, Linköping University, Linköping, Sweden; 3 Department of Oncology and Department of Biomedical and Clinical Sciences, Linköping University, Linköping, Sweden; 4 Department of Forensic Genetics and Forensic Toxicology, National Board of Forensic Medicine, Linköping, Sweden

**Keywords:** Breast cancer, Chemotherapy, Risk factors, Genetic testing, Translational research

## Abstract

Persistent taxane-induced peripheral neuropathy (TIPN) is highly prevalent among early-stage breast cancer survivors (ESBCS) and has detrimental effect on quality of life. We leveraged logistic regression models to develop and validate polygenic prediction models to estimate the risk of persistent PN symptoms in a training cohort and validation cohort taking clinical risk factors into account. Based on 337 whole-exome sequenced ESBCS two of five prediction models for individual PN symptoms obtained AUC results above 60% when validated. Using the model for *numbness in feet* (35 SNVs) in the test cohort, 73% survivors were correctly predicted. For *tingling in feet* (55 SNVs) 70% were correctly predicted. Both models included SNVs from the *ADAMTS20, APT6V0A2, CCDC88C, CYP2C8, EPHA5, NR1H3, PSKH2/APTV0D2*, and *SCN10A* genes. For *cramps in feet*, *difficulty climbing stairs* and *difficulty opening a jar* the validation was unsuccessful. Polygenic prediction models including clinical risk factors can estimate the risk of persistent taxane-induced *numbness in feet* and *tingling in feet* in ESBCS.

## Introduction

(Neo)adjuvant taxane chemotherapy contributes to favorable breast cancer prognosis but leaves 11%–80% of breast cancer survivors with persistent peripheral neuropathy (PN)^1,2^. Both prevention and treatment of PN are limited, and symptoms are associated with deterioration of health-related quality of life (QoL)^3–5^. Several clinical risk factors for PN have been identified: e.g., higher age, overweight, diabetes mellitus, smoking, previous neuropathy, cumulative dose, and treatment schedule^2,6,7^. Further paclitaxel is more prone to cause PN than docetaxel^7,8^. Nevertheless, some patients are not affected at all even if exposed to intensive treatment, suggesting variation in genetic predisposition.

Pharmacogenetic panel analysis to uncover genetic variation before treatment is under development. Recently a real-world study showed clinical utility of a 12-gene pharmacogenetic panel^9^ which identified actionable variants in >90% of the patients, suggesting that pharmacogenetics is both feasible and highly relevant. A decent proportion (30%) of clinically relevant drug-related adverse events were avoided. Two anticancer drug genes were on the panel—DPYD (5-FU) and UGT1A1 (irinotecan) —but in contrast to the 5-FU and irinotecan toxicity that in some cases can be predicted, no single gene has been identified as predictive of taxane-induced peripheral neuropathy (TIPN). Recently a systematic review and meta-analysis of TIPN pharmacogenetics concluded that few of identified genetic variants have been replicated in other studies, suggesting a complex combination of multiple genes and variants^10^. In the meta-analysis of 19 studies (6246 participants) including 60 single nucleotide variants (SNVs) in 23 genes, 13 SNVs (11 genes) were significantly associated with acute TIPN. The genes were mostly involved in liver metabolism and nerve function. TIPN is probably a polygenic trait, of which each gene has small-to-modest effects^11,12^. Broad genetic methods are needed to explore genetic variants with lower impacts. Few previous studies have used genome-wide or exome sequencing, and validation of polygenic prediction models of drug outcome is often lacking^10,13^.

In addition to a polygenic approach and broad genetic methods to identify the genotype, only a proportion of the drug-exposed can be expected to develop a toxicity phenotype, which leads to relatively small study populations in pharmacogenetic studies. The toxicity phenotype must also be clearly defined, clinically relevant and measured by standardized criteria^12^. PN symptoms are mostly physician-reported and studied as a summary score of sensory PN symptoms including numbness, tingling, and pain. However, PN can constitute both sensory and motor symptoms affecting function in different ways^5^. As there is no golden standard of measuring chemotherapy-induced PN (CIPN), studies have used different methods among which patient-reported validated instruments are considered more sensitive compared to physician-reported CIPN^14^.

We previously performed a population-based cross-sectional questionnaire study on 646 recurrence-free early-stage breast cancer (ESBC) survivors treated up to 7 years earlier with taxane chemotherapy^2^. By using the EORTC CIPN20 questionnaire^15^, we found an increased risk of 13 individual persistent sensory and motor PN symptoms when comparing survivors with matched female controls from the general population. These 13 persistent PN symptoms were associated with a significant and clinically relevant impact on global QoL, and functional health estimated by the EORTC QLQ C30 instrument^3^.

We hypothesize that the biological mechanisms and genetic background of different PN symptoms differ, and we therefore suggest that individual symptoms of TIPN need to be analyzed separately. In this substudy, we selected the five symptoms which, in our previous studies on persistent TIPN^2,3^, showed the largest increased risk compared to controls, and with the greatest impact on QoL, i.e., *numbness of toes and feet, tingling of toes and feet, cramps in feet, difficulty opening a jar or a bottle because of weakness in hands*, and *difficulty climbing stairs or getting up out of a chair because of weakness in legs*. The aim was to develop prediction models for these five persistent PN symptoms based on genetic and clinical risk factors. The genotype is based on whole-exome sequencing (WES) of the ESBC survivors. For validation of the developed prediction models, the study population was divided into a training cohort (*N* = 237) and a separate test cohort (*N* = 100) only used for validation.

## Results

Most survivors were treated with docetaxel (52.5%) and 3.0% had been exposed to both taxanes (Table 1). The proportion reporting moderate-severe symptoms varied between 12.1% and 27.6%. Survivor characteristics and TIPN symptom prevalence followed the same distribution as previously reported (data not shown)^2^. The training and test cohorts had no significant difference regarding age, BMI, diabetes mellitus, time since taxane treatment, and TIPN symptoms, however the test cohort had more often received paclitaxel treatment (<0.05).

**Table 1:** Survivor characteristics

	All (*N* = 337)	Missing data	Training cohort (*n* = 237)	Test cohort (*n* = 100)	*p*-value (Welch *t*-test)
Median age at survey, years (range)	62 (31–86)	0	62 (35–83)	62 (31-86)	0.922
Treatment N (%)					
Docetaxel	177 (52.5%)	0	132 (55.7%)	45 (45.0%)	0.074
Paclitaxel	150 (44.5%)	0	97 (40.9%)	53 (53.0%)	0.044
Both	10 (3.0%)	0	8 (3.4%)	2 (2.0%)	0.454
Mean dose mg/m^2^ (SD)					
Paclitaxel	842.4 (190.0)	0	833.0 (197)	860.4 (176.5)	0.044
Docetaxel	263.3 (60.5)	0	260.7 (66.9)	271.0 (34.4)	0.123
Mean BMI kg/m^2^ at survey (SD)	26.9 (4.7)	0	27.2 (4.6)	26.1 (4.7)	0.063
Treatment for diabetes mellitus N (%)	16 (4.7%)	0	11 (4.6%)	5 (5.0%)	0.890
Mean years from taxane to survey, (SD)	4.0 (1.5)	0	4.1 (1.6)	3.8 (1.5)	0.926
Proportion with moderate to severe taxane-induced peripheral neuropathy symptoms N (%)					
*Cramps in feet*	93 (27.6%)	2 (0.6%)	72 (30.1%)	21 (21.0%)	0.066
*Difficulty opening a jar*	88 (26.1%)	0	58 (24.5%)	30 (30.0%)	0.306
*Numbness in feet*	86 (25.5%)	2 (0.6%)	61 (25.7%)	25 (25.0%)	0.887
*Tingling in feet*	83 (24.6%)	2 (0.6%)	63 (26.6%)	20 (20.0%)	0.184
*Difficulty climbing stairs*	41 (12.1%)	0	28 (11.8%)	13 (13.0%)	0.766

### Sequencing output

Overall, the sequencing yielded on average 94.4 million unique reads/sample with an average median insert size of 181 base pairs, and an average GC-content of 49.4%. Of the reads 99.8% were aligned, yielding a final average exome target coverage of 74.7×, and on average 94.2% of the exome target was covered with ≥ 30×. After filtration of the VCF file, 174,330 high quality genetic variants were identified, of which 55,150 were common (minor allele frequency (MAF) ≥ 0.01). There were, on average, 19,383 non-reference high quality genetic variants per sample. The identity by descent (IBD) analysis showed no relationship or contamination of the samples (Supplementary Fig. S1A). Principal component analysis (PCA) based on identity by missingness (IBM) showed three samples outside of the cluster (Supplementary Fig. S1B). These three samples were also the ones with the most non-reference variants (Supplementary Fig. S1C), which explains their deviation in Fig. S1B, and therefore these three samples were still deemed reliable and were included in the remaining analyses. An analysis in ADMIXTURE for population stratification showed the lowest CV error for *K* = 1 at 0.15025 indicating a single population (Supplementary Fig. S4).

### Building and validation of prediction models

**Fig. 1:**
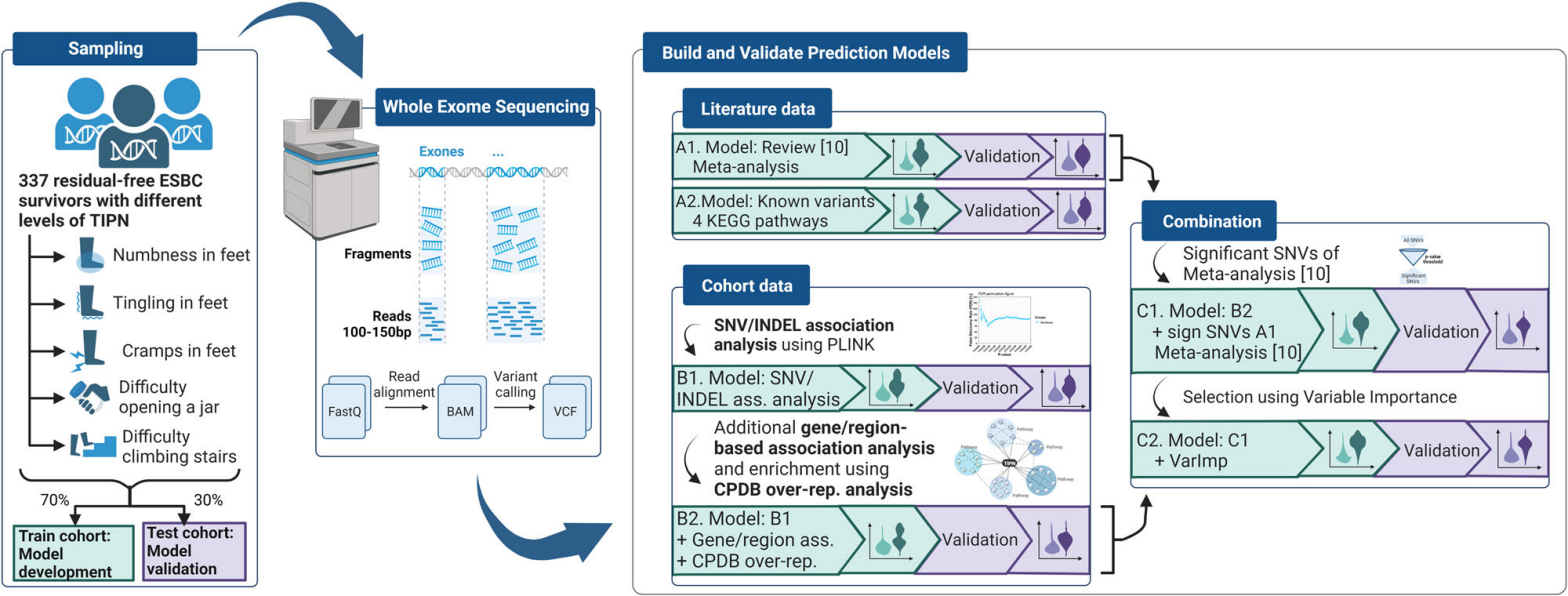
Flow chart of method design and prediction model development. The study was based on a sampling of 337 early-stage breast cancer survivors (ESBCS) with different levels of taxane-induced peripheral neuropathy (TIPN), divided into a training cohort (70%) and a test cohort (30%). Blood samples from the survivors were whole-exome sequenced. Building and validating logistic regression models was based on literature data, single nucleotide variants (SNVs) association analysis, gene/region association analysis, over-representation analysis, and Variable importance of the results from the whole-exome results and lastly by a combination. The figure was created in BioRender.com.

Prediction models were based on literature data/meta-analysis (models A1, A2), cohort data (single nucleotide variants (SNVs) association analysis, gene/region association analysis, pathway over-representation analysis) (models B1, B2) and lastly a combined model including Variable importance (models C1, C2), see Fig. 1.

#### Building and validation of prediction models based on previous meta-analysis

**Fig. 2:**
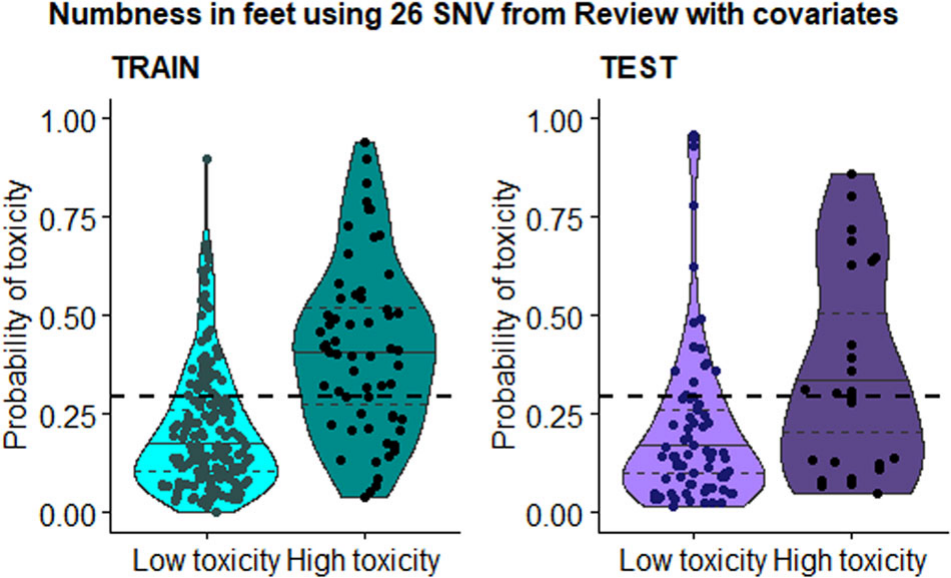
Probability of toxicity in the training and test cohort based on the A1 model for *numbness in feet.* Based on 26 SNVs from the meta-analysis^10^ and a cut-off at 0.29 (Table 2), 51% of those in the training cohort who were predicted to acquire high toxicity (above the cut-off) reported moderate-severe *numbness in feet*, and 47% in the test cohort. Of those with predicted to acquire low toxicity, 12% in training cohort and 16% in test cohort reported moderate-severe *numbness in feet*. The toxicity groups are based on the self-reported level of the symptom (EORTC CIPN20, low = None–A little, high = Quite a bit–Very much). Each dot in the violin plot is a prediction for a survivor.

Using all 60 SNVs in the meta-analysis by Guijosa et al.^10^, we found 26 SNVs in our WES data which were located or correlated to 18 genes including *ABCB1, EPHA5, CYP2C8*, and *SLCO1B1* (Supplementary Table S1). These 26 SNVs were included in the building of a logistic regression model in R. The performance of the models in each TIPN symptom were evaluated using AUC from the ROC curve where the AUC reached 72.06%–84.04% in the training cohort. In the validation run of the test cohort, the AUC for *numbness in feet* was 67.09% (Table 2) —however, for the remaining symptoms the AUC decreased to 32.8%–56.31%. The clinical risk factors improved the prediction for *numbness in feet* by increasing the AUC by 8.6% in the training cohort and 11.04% in the test cohort. Using the prediction model (A1) with a cut-off of 0.29 for *numbness in feet* resulted in a predicted high toxicity group (above the cut-off), where 47% of the patients in the test cohort reported persistent moderate-severe symptoms (51% in the training set), and a predicted low toxicity group, where 16% reported moderate-severe PN symptoms (12% in the training set) (Fig. 2). In total, 73% of the test cohort (training cohort 75%) were predicted correctly. The full model description for *numbness in feet* can be found in Supplementary Table S2. In these descriptions the reference allele is coded as 0, the heterozygote allele as 1 and the alternate allele as 2.

**Table 2:** Performance of prediction models A1 and C2 in taxane-induced peripheral neuropathy symptoms (EORTC CIPN20), including AUC of ROC curve (confidence interval 95%) and suitable cutoff for optimal accuracy, sensitivity, and specificity in both the training and test cohort

Symptoms	Model	FDR perm threshold	p (CPDB)	Review SNVs threshold	VarImp threshold	SNVs (genes)	Cutoff	Set	AUC (%) (CI 95%)	Accuracy (%)	Sensitivity (%)	Specificity (%)
***Numbness in feet***	A1	-	-	26	-	26 (16)	0.291	Train	78.74 (67-82)	75.32	70.49	77.01
								Test	**67.09 (54-78)**	**73.00**	**56.00**	**78.67**
***Tingling in feet***	A1	-	-	26	-	26 (16)	0.497	Train	75.42 (66-80)	79.57	33.33	96.51
								Test	*56.31 (41-71)*	*75.00*	*25.00*	*87.50*
***Cramps in feet***	A1	-	-	26	-	26 (16)	0.463	Train	72.06 (60-74)	75.32	33.33	93.87
								Test	*51.48 (36-64)*	*68.00*	*9.52*	*83.54*
***Difficulty opening a jar***	A1	-	-	26	-	26 (16)	0.411	Train	76.00 (67-81)	80.59	48.28	91.06
								Test	*55.71 (44-68)*	*63.00*	*20.00*	*81.43*
***Difficulty climbing stairs***	A1	-	-	26	-	26 (16)	0.394	Train	84.04 (60-79)	91.98	42.86	98.56
								Test	*32.80 (44-72)*	*78.00*	*0.00*	*89.66*
**Sum SNVs and genes**	**A1**	-	-	**26**	-	**26 (16)**	-	-	-	-	-	-
***Numbness in feet***	C2	0.006 (398)	0.0025	0.05 (8)	1.67	35 (40)	0.324	Train	88.87 (83-91)	83.40	77.05	85.63
								Test	**72.91 (60-84)**	**74.00**	**68.00**	**76.00**
***Tingling in feet***	C2	0.006 (408)	0.0190	0.05 (7)	1.2	55 (60)	0.346	Train	85.96 (79-90)	80.43	77.78	81.40
								Test	**60.88 (43-76)**	**69.00**	**50.00**	**73.75**
***Cramps in feet***	C2	0.007 (213)	0.1300	0.05 (8)	1.2	50 (57)	0.448	Train	85.69 (80-89)	80.43	65.28	87.12
								Test	*42.98 (41-70)*	*52.00*	*28.57*	*58.23*
***Difficulty opening a jar***	C2	0.007 (213)	0.1100	0.05 (8)	1.1	33 (42)	0.670	Train	80.34 (73-85)	78.06	15.52	98.32
								Test	*51.71 (40-65)*	*67.00*	*13.33*	*90.00*
***Difficulty climbing stairs***	C2	0.006 (213)	0.0600	0.05 (8)	1	35 (38)	0.644	Train	90.02 (82-93)	91.56	39.29	98.56
								Test	*42.88 (43-73)*	*75.00*	*0.00*	*86.21*
**Sum SNVs and genes**	**C2**	**(1119)**	-	**(8)**	-	**145 (158)**	-	-	-	-	-	-

Models performing AUC in test cohort above 60% in bold, AUC below 60% in italics.

In the A2 models however, the number of SNVs from the literature analyses were too large to build prediction models resulting in overfitting. However, 54 common SNVs could be extracted in the taxane pathway resulting in AUC results above 60% in the model building. Despite performance in the training cohort, the AUC from the ROC-curve only reached 47.33%–58.08% in the test set (Supplementary Table S3). These models were therefore not investigated further.

#### SNV association analysis, gene-based association analysis, and CPDB pathway over-representation analysis

The association analysis yielded, on average, 644 SNVs with a *p*-value ≤ 0.01 for the 5 TIPN symptoms, and of these on average 18.8%, 35.3%, and 45.9% were from the recessive, dominant, and additive logistic regressions models, respectively. By using permutations for evaluating the FDR (Supplementary Fig. S2) the *p*-value thresholds ≤0.0005–0.001125 for the 5 TIPN symptoms were chosen, yielding the lowest FDR of about 50%–80%. This resulted in a gain of 212 unique SNVs (13 SNVs overlap) and 234 unique genes for all TIPN symptoms (as defined by GRCh38.103.gtf) (note that some genetic variants cover multiple gene regions). Predictive logistic regression models based on the SNVs gained from these *p*-value thresholds were built. The models had an AUC of 72.55%–88.52% in the training cohort. SNV selection based on CADD score ≥ 13 (score for deleteriousness of SNVs and INDELs) did not improve the performance. The test cohort AUC reached only 40.14%–59.63%, and consequently these models were not investigated further (Supplementary Table S3).

For the gene tests, 19,337 unique genes were represented by at least two genetic variants, meaning that 173,372 unique genetic variants were mapped to genes and tested in the gene-based tests. Based on the Q–Q plots (Supplementary Fig. S3), the p-value threshold was set to ≤0.001. This yielded 134 unique genes (9 genes overlapped 2 symptoms) where 26, 31, 36, 29, and 21 genes were found for *numbness in feet*, *tingling in feet*, *cramps in feet*, *difficulty opening a jar*, and *difficulty climbing stairs*, respectively. Of the unique genes from the SNV association analysis, only LIPC was also found using the gene-based approach.

The gene tests results were combined with the wider inclusion of SNVs from the SNV/INDEL association analysis, which increased the coverage to ~1119 unique SNVs (163 SNVs overlap) and 971 unique genes. Using ~400 SNVs for *numbness in feet* and *tingling in feet*, 210 SNVs for *cramps in feet*, *difficulty opening a jar* and *difficulty climbing stairs*, with an enrichment using the over-representation analysis from CPDB of *p*-values < 0.005–0.2, the most optimal model building was found. The performance in AUC from the ROC curve gained 93.7%–98.67% in the training cohort. AUC decreased to 38.57%–50.31% in the test set for all but *numbness in feet*, reaching the maximum 68.43%, indicating that the prediction model optimization improved the models (Supplementary Table S3). These models were therefore optimized further and used in combination with the A1 models.

#### Combinational model of B2 and A1 using variable importance

Extracting all SNVs in the meta-analysis^10^ with *p*-values < 0.05 from previous statistical analysis, we found 8 SNVs (and 1 singularity): rs1056836 (*CYP1B1*), rs7349683 (*EPHA5*), rs1045642 (*ABCB1*), rs1128503 (*ABCB1*), rs10509681 (*CYP2C8*), rs1058930 (*CYP2C8*), rs8187710 (*ABCC2*), and rs1138272 (*GSTP1*) in our WES data. These 8 SNVs were added to the previous models constructed in B2, however rs1045642 (*ABCB1*) was already included in *tingling in feet*. Continuing with building of logistic regression models, a lower threshold of *p*-value < 0.0025–0.13 from the over-representation analysis in CPDB was needed for most models to avoid overfitting. This led to 260 unique SNVs (177 SNVs overlap), and 259 unique genes for all five TIPN symptoms, and gave the most optimal results. The models performed AUC in the ROC curve between 92.57%–98.05% in the training cohort, although in the test cohort the AUC decreased to 40.11%–52.34% for all except *numbness in feet*, where the AUC reached 71.15% (Supplementary Table S3). The clinical risk factors improved the model for *numbness in feet* by 8.36% in the training cohort, and by 2.08% in the test cohort. Nevertheless, the distribution of the prediction indicated that this model had a high risk of overfitting, and when efforts were made to avoid this AUC decreased rapidly in both the training and test cohorts and resulted in lower AUC results than the previous model in B2.

Using the VI gained from the previous model in C1, a threshold to obtain the most important variables receiving the highest AUC in the training cohort were set between 1.00–1.67. This led to 35 SNVs (40 unique genes) for *numbness in feet*, 55 SNVs (60 unique genes) for *tingling in feet*, 50 SNVs (57 unique genes) for *cramps in feet*, 33 SNVs (42 unique genes) for *difficulty opening a jar*, 35 SNVs (38 unique genes) for *difficulty climbing stairs*. The model performed an AUC from the ROC-curve at 80.34%–90.02% in the training cohort, and 42.98%–72.91% in the test cohort, with *numbness in feet* and *tingling in feet* above 60% (Table 2, Supplementary Table S3).

**Fig. 3:**
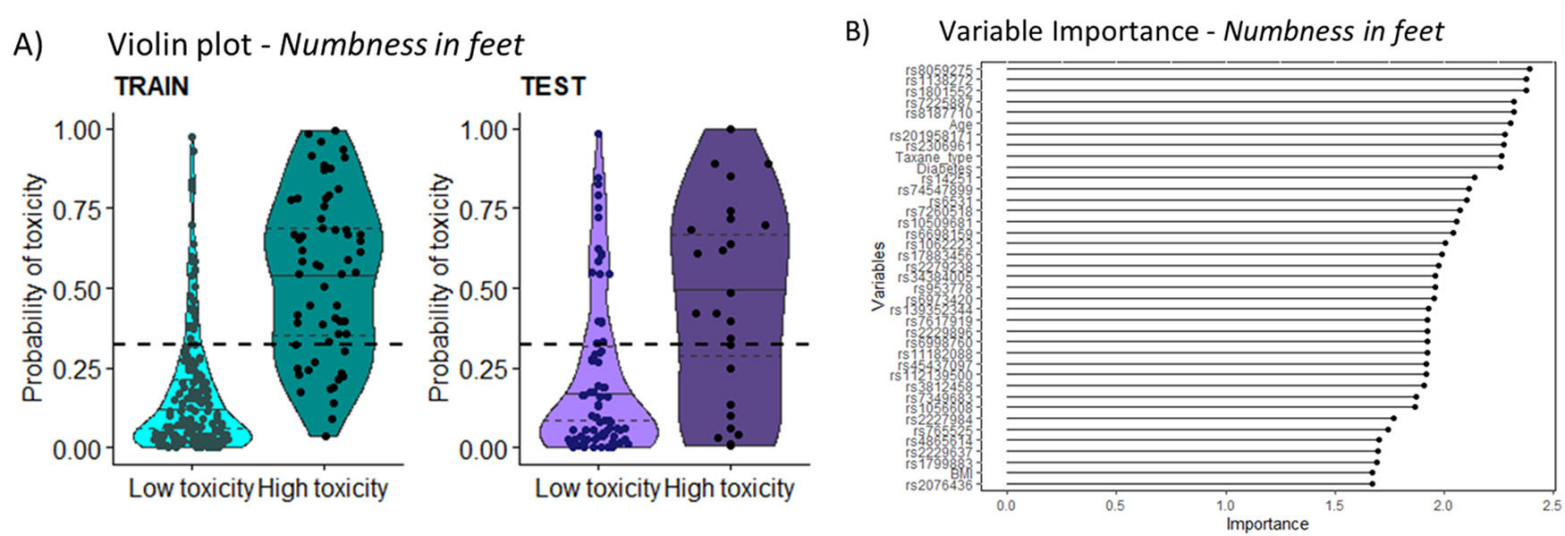
Probability of toxicity and Variable importance for the C2 model in the training and test cohort for *numbness in feet.* The toxicity groups are based on the EORTC CIPN20, low = None–A little, high = Quite a bit–Very much. Each dot is a prediction for a survivor. The included SNVs and corresponding genes can be seen in Supplementary Table S1. **A** Using a cut-off at 0.324 (Table 2), 65% of those in the training cohort who were predicted to acquire high toxicity (above the cut-off) reported moderate-severe *numbness in feet*, and 47% in the test cohort. Of those with predicted to acquire low toxicity, 8% in training cohort and 14% in test cohort reported moderate-severe *numbness in feet*. **B** The Variable importance (VI) plot shows and their VI based on the previous model in C1. The VI plot for *numbness in feet* includes 35 SNVs and 4 clinical risk factors.

**Fig. 4:**
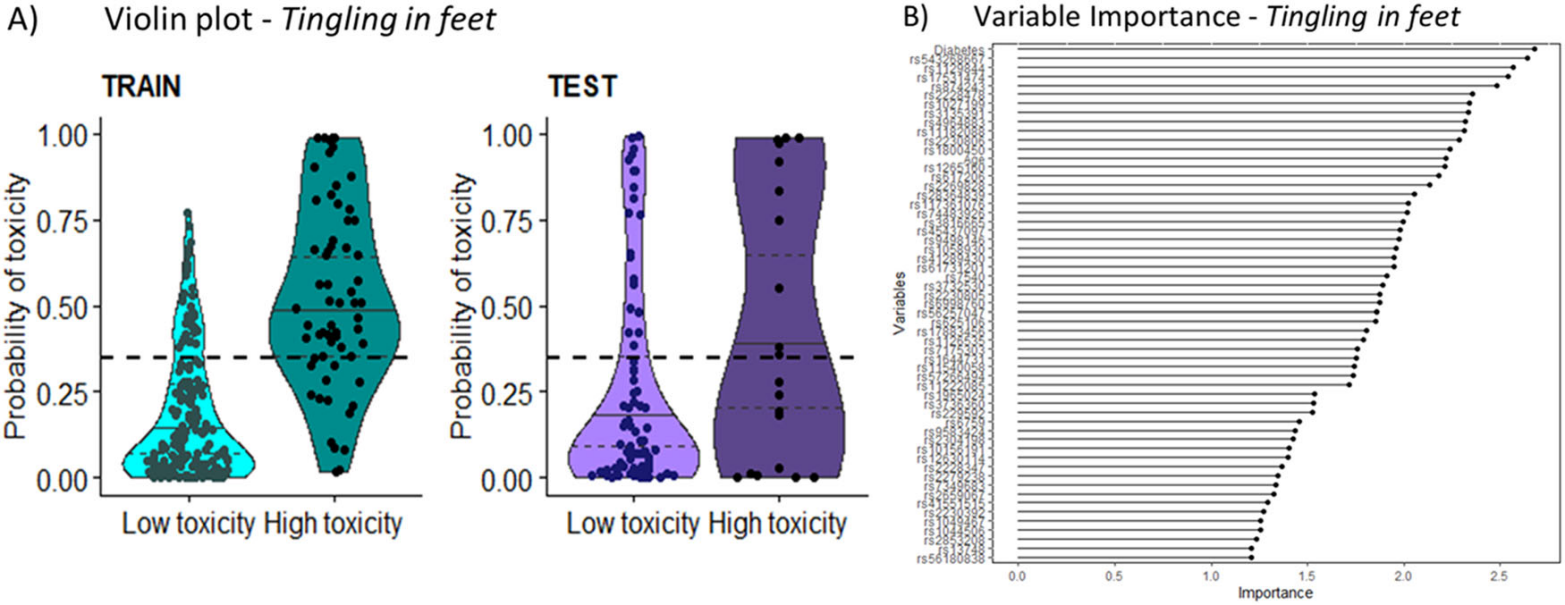
Probability of toxicity and Variable importance for the C2 model in the training and test cohort for *tingling in feet.* The toxicity groups are based on the EORTC CIPN20, low = None–A little, high = Quite a bit–Very much. Each dot is a prediction for a survivor. The included SNVs and corresponding genes can be seen in Supplementary Table S1. **A** Using a cut-off at 0.346 (Table 2), 60% of those in the training cohort who were predicted to acquire high toxicity reported moderate-severe *tingling in feet*, and 33% in the test cohort. Of those with predicted to acquire low toxicity, 9% in training cohort and 14% in test cohort reported moderate-severe *tingling in feet*. **B** The Variable Importance (VI) plot shows the included variables and their VI based on the previous model in C1. VI plot for *tingling in feet*: 55 SNVs and 2 clinical risk factors.

Using the prediction model C2 for *numbness in feet* with a cut-off of 0.32 resulted in a predicted high toxicity group above the cut-off, where 47% of the survivors in the test cohort reported persistent moderate-severe *numbness in feet* (65% in the training cohort), and a predicted low toxicity group, where 14% reported moderate-severe symptoms (training cohort 8%) (Fig. 3). In total, 73% of the test cohort (training cohort 83%) were predicted correctly. Using the prediction model C2 for *tingling in feet* with a cut-off 0.35 resulted in a predicted high toxicity group above the cut-off, where 33% of the survivors reported moderate-severe *tingling in feet* (training cohort 60%), and a predicted low toxicity group, where 14% reported persistent moderate-severe symptoms (training cohort 9%) (Fig. 4). In total, 70% of the test cohort (training cohort 82%) were predicted correctly. Full models with estimates/regression coefficients, confidence intervals, standardized errors, confusion matrix and *p*-values for each variable can be found in the Supplementary Table S2.

The SNVs included in the models are shown in Figs. 3 and 4 but are also listed with frequencies and corresponding genes in Supplementary Table S1. In both symptoms, the SNVs rs11182088 *(ADAMTS20)*, rs17883456 *(APT6V0A2)*, rs45437097 *(CCDC88C)*, rs7349683 *(EPHA5)*, rs2279238 *(NR1H3)*, and rs6998760 *(APTV0D2; PSKH2)*, with the additional genes *CYP2C8* (rs10509681 in *numbness in feet* and rs1058930 in *tingling in feet*) and *SCN10A* (rs7617919 in *numbness in feet* and rs57326399 in *tingling in feet*) were found. The SNVs for the genes *ABCC2*, *CYP2C8*, *EPHA5, GSTP-1* were from the meta-analysis^10^. The other genes are correlated to the Methadone action pathway (*p*-value = 0.000104) and Axon guidance pathway (*p*-value = 0.00979) based on CPDB. Comparison of pathways in CPDB assays revealed that the genes in the models for *numbness in feet* and *tingling in feet* in C2 had two pathways in common with the meta-analysis^10^ genes included in the A1 models; Receptors in lipid metabolism and toxicity and Nuclear receptors meta-pathway (*p*-values < 0.01.). The full results of the CPDB assay can be found in Supplementary Table S4.

## Discussion

Previous polygenic prediction models to estimate risk for persistent taxane-induced peripheral neuropathy are few and have not been validated. In our study, polygenic prediction models based on whole-exome sequencing of 337 ESBC survivors, SNVs from a previous literature meta-analysis, and clinical risk factors were developed and then validated in a test cohort. The final prediction models could distinguish between high and low risk for two PN symptoms, i.e., *numbness in feet* and *tingling infeet*. For *cramps in feet*, *difficulty opening a jar* and *difficulty climbing stairs* the prediction models could not be validated, suggesting a more complex biological background. Identification of different risk groups may help clinicians to tailor adjuvant treatment for early-stage breast cancer patients.

The final prediction models were built using SNVs selected by a data driven false discovery rate for each individual symptom, followed by pathway/network enrichment, and optimization based on Variable importance and the inclusion of previously associated SNVs, each step increasing accuracy of the variants and weight of each variable. Using our final model (C2) for *numbness in feet*, a total of 73% of the test cohort were correctly classified in the validation. For *tingling in feet*, 70% survivors of the test cohort were predicted correctly. Should our models be validated in external cohorts, we would clinically identify at a group of patients with a probability of persistent *numbness in feet* and *tingling in feet* of 47% respectively 33% compared to a low-risk group with only 14% toxicity. Reported neuropathy expected in a low-risk group, since a prevalence of 7.9%–11.1% has been reported in the general population^16^. Specifically for the symptoms *numbness in feet* and *tingling in feet*, moderate-severe neuropathy was reported by 7.2% respectively 6.7% among matched female controls without cancer^2^.

To the best of our knowledge, only one previous study included a prediction model for TIPN symptoms. Based on a GWAS of 183 breast and ovarian cancer patients, Hooshmand et al. presented a polygenic risk score (PRS) of 46 SNVs that significantly correlated with a summary score of all scales (CIPN20)^17^. Another approach, used to identify TIPN risk by GWAS is a hierarchical SNP cluster of 267 SNPs that could predict CIPN toxicity in 603 breast cancer patients with either none or grade 3–4 CIPN (Common Terminology criteria for adverse events (CTCAE) scale)^18^. Compared to these two studies, we have validated the models in a separate test cohort using a random split internal validation method^19^. We aimed to include a limited number of predictors (33–55 SNVs) to avoid models overfitting the training cohort and not performing in the subsequent test cohort. All models performed AUC > 80% for all symptoms in the training cohort but for three symptoms the models failed to show AUC > 60% in the test cohort, showing the importance of validating these types of model developments.

We used the EORTC CIPN20 instrument to assess self-reported TIPN in line with the recommendation by NCI^20^. Consistent phenotype definition is fundamental for the accuracy of results^21^. Interestingly, the identified SNVs with genome-wide significance in Hooshmand’s study were associated with patient-reported neuropathy but no other neuropathy outcome measures e.g., NCI-CTCAE, which might indicate the need to be more accurate when assessing the TIPN phenotype. We therefore used individual self-reported PN symptoms, which provide more clearly defined toxicity phenotypes in our study compared to most previous studies, which in turn enables investigation of associations with different pathophysiological mechanisms. Sensory symptoms like tingling or pain in hands/feet are caused by an increased number of ion channels, altered calcium signaling, neuroinflammation, and activation of nociceptors, whereas the symptoms of numbness and decreased proprioception in hands/feet are caused by loss of intradermal nerve fibers, demyelinization, and nerve degeneration^5,22^. In our prediction models, clinical risk factors had varying importance depending on the individual TIPN symptom. This difference may indicate that various pathophysiological and pathogenetic mechanisms are involved for the individual PN symptoms. For example, all clinical risk factors were of high importance for predicting *numbness in feet*. For *tingling in feet*, diabetes mellitus treatment was of high importance, age was relatively important, and paclitaxel and BMI were of lower importance. Although not included in this study, biomarkers associated with increased risk for CIPN such as p16 indicating aging and cellular senescence, could possibly also improve future models^23^.

We identified the Methadone action pathway, Axon guidance pathway, Receptors in lipid metabolism and toxicity, and Nuclear receptors meta-pathway to be important in TIPN, similar to drug metabolism, axon development and regeneration pathways reported by others^10,17,18^. For example, one gene in the prediction models in our study is EPHA5, an EPHA gene crucial for nervous system development, tissue regeneration, and in ephrin-A signaling^24,25^. EPHA5 is well-known to be associated with TIPN and has been independently replicated by several others^17,26^. We compared the exome-located variants in Hooshmand’s PRS^17^ and Lustberg’s SNV cluster^18^ and found no overlap with the SNVs in our prediction models. As expected, many of the SNVs included in our final models have not been published before in relation to TIPN, which is not surprising, since most (98%) significant genetic variants in pharmacogenetic GWAS studies are unidentified in previously studies^27^.

In the final prediction models (C2) for persistent *numbness in feet* and *tingling in feet*, three genes/gene families from A1 were included in both models: EPHA5 (see above), ABC family, and *CYP2C8*. The ABC genes are involved in drug metabolism through active efflux pumps for cellular clearance and excretion. *CYP2C8* is important in paclitaxel metabolism, and the included variants are associated with low activity^28^. *GSTP-1*, also a drug metabolism gene from the A1 model, was included only in the C2 model for *numbness in feet*. In addition to these previously established TIPN genes, six genes were included in both our final models, suggesting that these are genes that are common for the two symptoms. First, *SCN10A* from a family of voltage-gated sodium channels involved in pathogenesis of neuropathic pain^29^, idiopathic and diabetes neuropathy^30^. Association between *SCN9A*, but not *SCN10A*, and CIPN has previously been published^31^. The second gene *CCDC88C* has no documented association with neuropathy, but *CCDC121* in the same family has been associated with cytotoxicity and TIPN^32^. Third, for the *NR1I3* gene, another SNV has been reported to be protective against CIPN^33^ in a small study of CIPN and genes related to absorption, distribution, metabolism, and excretion of drugs in breast cancer patients. To the best of our knowledge, for the fourth and fifth genes—*ATP6V0A2* and *ATPV0D2; PSKH2*—no association with neuropathy has yet been found. In both models SNVs in genes from the ADAMTS family had the highest Variable importance, *ADAMTS18* for *numbness in feet* and *ADAMTS7* for *tingling in feet. ADAMTS20* is included in both models but to our knowledge not previously reported in association with peripheral neuropathy. The ADAMTS gene family members are extracellular protease enzymes associated for example with tissue remodeling and evidence indicate that ADAMTS play an important role in neuroplasticity as well as nervous system pathologies^34^. The role of ADAMTS gene family in relation to PN merits further exploration.

The strengths of this study are the broad genetic method, the patient-reported phenotype, and the size of the cohort of survivors exposed to chemotherapy in accordance with international guidelines and the use of a validation cohort. The degree of missing data is low. By studying the symptoms separately, the phenotype is well-defined. We have validated our own results by dividing the study population into a training cohort and a separate test cohort. Possible limitations in this study are the absence of information on some risk factors such as pre-existing neuropathy and both BMI and diabetes mellitus treatment was self-reported. Due to the absence of data on persistent TIPN phenotype, we used the SNVs from Guijosa’s meta-analysis which is based on acute TIPN phenotype. We cannot rule out that there may be differences in acute and persistent TIPN due to the absence of data on persistent TIPN. The prediction models for three symptoms could not be validated, possibly due to a more multifactorial background at least for *difficulty opening a jar* and *difficulty climbing stairs*. The study is based on exome sequencing (protein-coding DNA), and therefore 34 non-coding SNVs from the recent meta-analysis could not be included in our models^10^. The genetic predictors found may only be valid within the specific population studied. Future validation in external cohorts will be needed as the risk of overfitting is a general limitation in prediction modelling. The test cohort consisted of only 100 survivors and differed somewhat from the training cohort in that fewer received paclitaxel.

Overall, our results provide a proof-of-concept towards personalized risk estimation of persistent individual TIPN symptoms and may enable a risk-benefit assessment of (neo-) adjuvant taxane treatment in early-stage breast cancer for clinical use. A genetic high susceptibility to persistent TIPN may play a role in drug selection depending on risk-benefit assessment and patient preference and may lead to e.g., increased surveillance during taxane treatment. The polygenic risk model supported by clinical risk factors predicted the symptoms *numbness in feet* and *tingling in feet*, emphasizing the importance of a well-defined toxicity phenotype by separating individual TIPN symptoms before pharmacogenetic prediction can be achievable.

## Methods

**Fig. 5:**
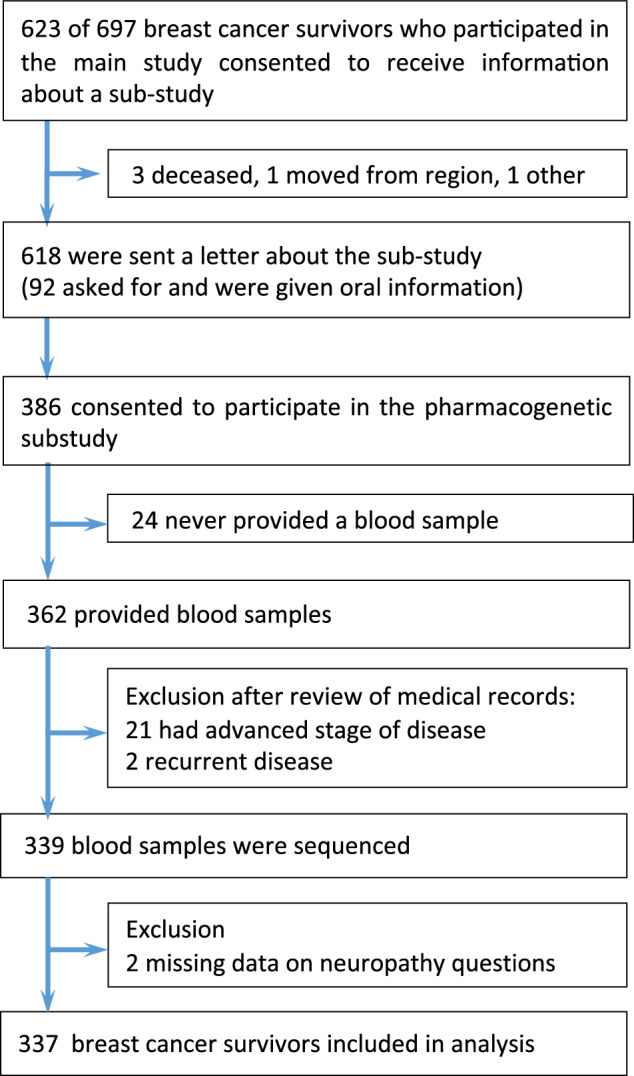
Flow chart of study population.

The study population is a subset from our previous study^2^ (Fig. 5) of survivors previously treated with (neo)adjuvant taxane-containing therapies for ESBC diagnosed between January 2010 and June 2015 in the Southeast Health Care region, Sweden who received a postal questionnaire including the validated EORTC QLQ-C30 and CIPN20 instruments. The study was cross-sectional and peripheral neuropathy is self-reported in median 3.6 years after taxane. Details on chemotherapy were obtained from the CSAM Cytodos software system, while body mass index (BMI) and diabetes mellitus treatment were self-reported at the survey^2^.

One year after answering the survey 623 respondents received an invitation letter to the genetic substudy and had the possibility to receive additional information by telephone from a research nurse or doctor. A written informed consent was provided by all study participants. Blood samples were collected over 4 months. Finally, 337 of 362 survivors who provided blood samples were included in the analysis (Fig. 5). The study received ethical approval from the regional ethics committee in Linköping, Sweden (DNR 2018/94-32). This study was conducted in accordance with the principles of the Declaration of Helsinki, and all patients provided written informed consent prior to enrollment.

The TIPN symptoms; *numbness in feet, tingling in feet, cramps in feet, difficulty opening a jar*, and *difficulty climbing stairs* were dichotomized into survivors reporting “quite a bit” or “very much” of the symptom (i.e., moderate-severe symptoms) and survivors reporting “not at all” or “a little”. Before association testing, the survivors were split up into one training cohort (70%, *n* = 237) and a separate test cohort (30%, *n* = 100) only used for validation (Fig. 1).

### Whole-exome sequencing

DNA was extracted from peripheral whole blood samples using the Maxwell® 16 Instrument (Promega, Madison, WI, USA) and the Maxwell® 16 Blood DNA Purification Kit (Promega) according to the manufacturer’s protocol. Sequencing libraries were prepared from 50 ng of DNA per sample using the Twist Human Core Exome sample preparation kit (Twist Bioscience, South San Francisco, CA, USA,) with unique dual indexes. Sequencing was performed by the accredited laboratory SNP&SEQ Technology Platform at Uppsala University (Sweden) on a NovaSeq 6000 Sequencing System (Illumina, San Diego, CA, USA) using NovaSeq 6000 S4 and SP flowcells with v1 sequencing chemistry.

The FASTQ-files with raw sequencing reads were aligned to the human reference genome (GRCh38) using the aligner BWA version 0.7.17^35,36^. The resulting BAM-files were de-duplicated with Picard (http://broadinstitute.github.io/picard/) MarkDuplicates. Recalibration was done using Genome Analysis Toolkit (GATK) version 4.1.0.0^37–39^ BaseRecalibrator and GATK ApplyBQSR. SNVs and insertion-deletion mutations (INDELs) were then called using GATK HaplotypeCaller in GVCF-mode before they were combined and jointly genotyped using GATK CombineGVCFs and GATK GenotypeGVCFs. After this, variant quality scores were recalibrated with GATK VariantRecalibrator and GATK ApplyVQSR. Lastly, the genetic variants were annotated using Combined Annotation Dependent Depletion (CADD) version GRCh38-v1.6^40^. During the alignment and variant calling process, quality metrics were collected with FastQC version 0.11.8, SAMtools version 1.8^41^, BCFtools version 1.10, and Qualimap version 2.2.2^42^. All quality metrics were summarized and analyzed using MultiQC version 1.7^43^. VCFtools version 0.1.16^44^ was used to remove genetic variants with a genotyping rate <0.95, a mean coverage < 10 across all samples, and a Hardy-Weinberg *p* < 0.0001 that were not bi-allelic, or not labelled with PASS.

### Building and validating prediction models

For building and validation of prediction models, *R* version 4.0.3^45^ was used. All models were built through logistic regression with binominal distribution using the function glm in the R package stats (version 3.6.2). The first model was based on the recent meta-analysis of variants and genes associated with TIPN^10^ (A1 in Fig. 1) where the most significant variants using *p*-value < 0.05 were included in our models. Building of the second model continued with sets of variants and genes previously associated with TIPN and the four pathways important for the therapy^46^ or for neuropathy^47^ (A2 in Fig. 1).

Secondly, model building was based on the WES cohort data included in this substudy. The models were based on a SNV/INDEL association analysis for each TIPN symptom using PLINK version 1.90b4.9^48^ established with logistic regression (additive, dominant, or recessive). Using 1000 permutations to find the lowest false discovery rate (FDR) for each TIPN symptom, variants below a symptom specific *p*-value (*p*-values in the range 0.00001–0.005 were tested) in the permutations were extracted to the model building (B1 in Fig. 1). The next model continued with the SNV/INDEL association analysis including 200-450 SNVs, and a gene/region association analysis of rare SNVs/INDELs using R-package SKAT^49,50^. For SNV selection in the models (B2 in Fig. 1) over-representation analysis in ConsensusPathDB-human (CPDB) Release 35 (05.06.2021)^51^ based on the pathway correlations in the found variants and genes were used.

Finally, the B2 models were combined with the SNVs from the A1 models, and the *p*-value threshold based on CPDB analyses were also adjusted resulting in the C1 in Fig. 1. Next Variable Importance (VI) for each variable was collected from the C1 model with the R function glm in the R package stats (version 3.6.2). The highest ranked SNVs in VI were selected and included in the C2 models (Fig. 1). The clinical risk factors taxane type and BMI were also included in the model for *tingling in feet* due to previous findings^2^ despite lower VI. More information about each model building is provided in Supplementary Material S1.

The models were built using the training cohort, and missing data for individual TIPN symptom were excluded. Age, taxane type, BMI, and treatment for diabetes mellitus were added as covariates and missing data were handled as default in R. Singularities of SNVs found by the logistic regression building, indicating of two or more SNVs with an exact linear relationship, were excluded to ensure that the ordinary least squares estimate of the regression parameters would be unique. The models with the best predicting capacity were determined primarily by evaluating the receiver operating characteristic (ROC) area under the curve (AUC) for the training data, and secondly by evaluating the accuracy, sensitivity, and specificity of the model. These models were thereafter validated in the test cohort. The final prediction model was considered optimal when reaching an AUC in the training cohort above 80% without creating an overfitted model and an AUC above 60% in the separate test cohort. Cut-offs for optimal models were adjusted to give an accuracy around 80%.

### Reporting summary

Further information on research design is available in the Nature Research Reporting Summary linked to this article.

### Supplementary information

Supplementary material

REPORTING SUMMARY

## Data Availability

The data generated in this study are available upon reasonable request from the corresponding author. Unfortunately, the ethical approval does not allow for the sequencing data to be deposited into a secure access-controlled repository.

## Code availability

We utilized freely available open-source functions and programs all referred to along with the specific version numbers indicated in the Methods section. The custom scripts are available at https://github.com/huvdal/Prediction_of_TIPN.git.

## References

[1] Rivera, D. R., Ganz, P. A., Weyrich, M. S., Bandos, H. & Melnikow, J. Chemotherapy-associated peripheral neuropathy in patients with early-stage breast cancer: a systematic review. *J.**Natl Cancer Inst.* **110**, djx140 (2018). 10.1093/jnci/djx140 PMC5825681 28954296

[2] Engvall, K., Gréen, H., Fredriksson, M. & Åvall-Lundqvist, E. Persistent neuropathy among early-stage breast cancer survivors in a population-based cohort. *Br J. Cancer* **125**, 445–457 (2021). 10.1038/s41416-021-01429-3 PMC8329002 34017086

[3] Engvall K (2022). Impact of persistent peripheral neuropathy on health-related quality of life among early-stage breast cancer survivors: a population-based cross-sectional study. Breast Cancer Res. Treat..

[4] Loprinzi CL (2020). Prevention and management of chemotherapy-induced peripheral neuropathy in survivors of adult cancers: ASCO guideline update. J. Clin. Oncol..

[5] Jordan B (2020). Systemic anticancer therapy-induced peripheral and central neurotoxicity: ESMO-EONS-EANO clinical practice guidelines for diagnosis, prevention, treatment and follow-up. Ann. Oncol..

[6] Greenlee H (2017). BMI, lifestyle factors and taxane-induced neuropathy in breast cancer patients: the pathways study. J. Natl Cancer Inst..

[7] Bao T (2016). Long-term chemotherapy-induced peripheral neuropathy among breast cancer survivors: prevalence, risk factors, and fall risk. Breast Cancer Res. Treat..

[8] Sparano JA (2008). Weekly paclitaxel in the adjuvant treatment of breast cancer. N. Engl. J. Med..

[9] Swen JJ (2023). A 12-gene pharmacogenetic panel to prevent adverse drug reactions: an open-label, multicentre, controlled, cluster-randomised crossover implementation study. Lancet.

[10] Guijosa A (2022). Pharmacogenetics of taxane-induced neurotoxicity in breast cancer: systematic review and meta-analysis. Clin. Transl. Sci..

[11] McInnes G, Yee SW, Pershad Y, Altman RB (2021). Genomewide association studies in pharmacogenomics. Clin. Pharm. Ther..

[12] Church D (2014). Toxgnostics’: an unmet need in cancer medicine. Nat. Rev. Cancer.

[13] Siemens, A., Anderson, S. J., Rassekh, S. R., Ross, C. J. D. & Carleton, B. C. A systematic review of polygenic models for predicting drug outcomes. *J. Pers Med.* **12**, 1394 (2022). 10.3390/jpm12091394 PMC9505711 36143179

[14] Tan AC, McCrary JM, Park SB, Trinh T, Goldstein D (2019). Chemotherapy-induced peripheral neuropathy-patient-reported outcomes compared with NCI-CTCAE grade. Support Care Cancer.

[15] Postma TJ (2005). The development of an EORTC quality of life questionnaire to assess chemotherapy-induced peripheral neuropathy: the QLQ-CIPN20. Eur. J. Cancer.

[16] Hanewinckel R (2016). Prevalence of polyneuropathy in the general middle-aged and elderly population. Neurology.

[17] Hooshmand K (2022). Polygenic risk of paclitaxel-induced peripheral neuropathy: a genome-wide association study. J. Transl. Med..

[18] Lustberg M (2023). Identification of a SNP cluster associated with taxane-induced peripheral neuropathy risk in patients being treated for breast cancer using GWAS data derived from a large cooperative group trial. Support Care Cancer.

[19] Dankers, F., Traverso, A., Wee, L. & van Kuijk, S. M. J. In *Fundamentals of Clinical Data Science* ((eds) P. Kubben, M. Dumontier, & A. Dekker) 101–120 (Springer Copyright 2019, The Author(s). 2019).

[20] Dorsey SG (2019). The national cancer institute clinical trials planning meeting for prevention and treatment of chemotherapy-induced peripheral neuropathy. J. Natl Cancer Inst..

[21] Cliff J (2017). The molecular genetics of chemotherapy-induced peripheral neuropathy: a systematic review and meta-analysis. Crit. Rev. Oncol. Hematol..

[22] Laforgia, M. et al. Peripheral neuropathy under oncologic therapies: a literature review on pathogenetic mechanisms. *Int. J. Mol. Sci.* **22**, 1980 (2021). 10.3390/ijms22041980 PMC7922628 33671327

[23] Mitin N (2022). A biomarker of aging, p16, predicts peripheral neuropathy in women receiving adjuvant taxanes for breast cancer. NPJ breast cancer.

[24] Miao H, Wang B (2012). EphA receptor signaling-complexity and emerging themes. Semin Cell Dev. Biol..

[25] Baldwin RM (2012). A genome-wide association study identifies novel loci for paclitaxel-induced sensory peripheral neuropathy in CALGB 40101. Clin. Cancer Res..

[26] Leandro-Garcia LJ (2013). Genome-wide association study identifies ephrin type A receptors implicated in paclitaxel induced peripheral sensory neuropathy. J. Med. Genet.

[27] Linskey DW, Linskey DC, McLeod HL, Luzum JA (2021). The need to shift pharmacogenetic research from candidate gene to genome-wide association studies. Pharmacogenomics.

[28] Marcath LA (2019). Patients carrying CYP2C8*3 have shorter systemic paclitaxel exposure. Pharmacogenomics.

[29] Faber CG (2012). Gain of function Naν1.7 mutations in idiopathic small fiber neuropathy. Ann. Neurol..

[30] Wadhawan S (2017). Na(V) channel variants in patients with painful and nonpainful peripheral neuropathy. Neurol. Genet.

[31] Tanabe Y (2020). Taxane-induced sensory peripheral neuropathy is associated with an SCN9A single nucleotide polymorphism in Japanese patients. BMC Cancer.

[32] Komatsu M (2015). Pharmacoethnicity in paclitaxel-induced sensory peripheral neuropathy. Clin. Cancer Res..

[33] Arbitrio M (2019). Polymorphic variants in NR1I3 and UGT2B7 predict taxane neurotoxicity and have prognostic relevance in patients with breast cancer: a case-control study. Clin. Pharm. Ther..

[34] Gurses MS, Ural MN, Gulec MA, Akyol O, Akyol S (2016). Pathophysiological function of ADAMTS enzymes on molecular mechanism of alzheimer’s disease. Aging Dis..

[35] Li H, Durbin R (2009). Fast and accurate short read alignment with Burrows-Wheeler transform. Bioinformatics.

[36] Li H, Durbin R (2010). Fast and accurate long-read alignment with Burrows-Wheeler transform. Bioinformatics.

[37] DePristo MA (2011). A framework for variation discovery and genotyping using next-generation DNA sequencing data. Nat. Genet.

[38] McKenna A (2010). The genome analysis toolkit: a MapReduce framework for analyzing next-generation DNA sequencing data. Genome Res..

[39] Van der Auwera, G. A. et al. From FastQ data to high confidence variant calls: the genome analysis toolkit best practices pipeline. *Curr. Protoc. Bioinformatics* **43**,11.10.1-11.10.33 (2013). 10.1002/0471250953.bi1110s43 PMC4243306 25431634

[40] Rentzsch P, Witten D, Cooper GM, Shendure J, Kircher M (2019). CADD: predicting the deleteriousness of variants throughout the human genome. Nucleic Acids Res..

[41] Li H (2009). The sequence alignment/Map format and SAMtools. Bioinformatics.

[42] García-Alcalde F (2012). Qualimap: evaluating next-generation sequencing alignment data. Bioinformatics.

[43] Ewels P, Magnusson M, Lundin S, Käller M (2016). MultiQC: summarize analysis results for multiple tools and samples in a single report. Bioinformatics.

[44] Danecek P (2011). The variant call format and VCFtools. Bioinformatics.

[45] RCoreTeam, R. *A Language and Environment for Statistitical Computing*. https://www.r-project.org/ (2021).

[46] Oshiro C (2009). Taxane pathway. Pharmacogenet Genom..

[47] Kanehisa M, Goto S (2000). KEGG: kyoto encyclopedia of genes and genomes. Nucleic Acids Res..

[48] Purcell S (2007). PLINK: a tool set for whole-genome association and population-based linkage analyses. Am. J. Hum. Genet.

[49] Ionita-Laza I, Lee S, Makarov V, Buxbaum JD, Lin X (2013). Sequence kernel association tests for the combined effect of rare and common variants. Am. J. Hum. Genet.

[50] Lee S, Wu MC, Lin X (2012). Optimal tests for rare variant effects in sequencing association studies. Biostatistics.

[51] Herwig R, Hardt C, Lienhard M, Kamburov A (2016). Analyzing and interpreting genome data at the network level with ConsensusPathDB. Nat. Protoc..

