# Supplementary material for: Prediction models of persistent taxane-induced peripheral neuropathy among breast cancer survivors using whole-exome sequencing
Source: NPJ Precis Oncol. 2024 May 16;8:102. doi: 10.1038/s41698-024-00594-x (PMC11099113; doi:10.1038/s41698-024-00594-x)
Supplement: Supplementary file 1 — Supplementary material [file 41698_2024_594_MOESM1_ESM.pdf]

Supplementary SM1 Method description

A1: Variants based on previous meta-analysis

Based on a recent meta-analysis[1] , variants previously associated with taxane-induced neuropathy were included in a model.

A2: Model based on predefined and previously known SNVs and genes

We also investigated sets of variants (rs209709, rs5749248, rs1753097, rs4737264, rs7349683, rs301927, rs17348202, rs1159057, rs10771973, and rs3125923) and genes (*ERCC1, EPHA4, EPHA5, EPHA6, EPHA8, XKR4, FZD3, XPC, LIMK2, PIK3IP1, SLCO1B3, SGCG, HIF1A, ABCB1, ABCG2, NQO1, CYP3A4, CYP3A5, CYP2C8, and TUBB2A)* previously associated with taxane-induced neuropathy and genes, and variants present in four pathways important for therapy, via the taxane pathway[2] , or neuropathy, via the KEGG [3] pathways: axon guidance (KEGG entry: map04360), regeneration (KEGG entry: map04361), and neurodegeneration (KEGG entry: map05022).

B1: Model based on cohort data using SNV/INDEL association analysis

A SNV/INDEL association analysis was used to find suitable variants to include in the first cohort data model, B1. PLINK version 1.90b4.9 [4] was used to compute identity-by-descent (IBD) and identity-by-missingness (IBM) metrics. PLINK was also used for association testing of common SNVs/INDELs (minor allele frequency (MAF) ≥1%) and the five case/control TIPN phenotypes using additive, dominant, and recessive genetic logistic regression models adjusted for the covariates age at survey (years), taxane type (docetaxel, paclitaxel, or both), docetaxel dose (mg/m^2), paclitaxel dose (mg/m^2), time since treatment (months), body mass index (BMI) at survey (kg/m^2), and diabetes (yes or no).

For each genetic variant and toxicity phenotype, the logistic regression model (additive, dominant, or recessive) with the lowest p-value was selected. 1000 permutations with randomly and uniquely shuffled toxicity phenotype values were used to determine at what p-value threshold the true toxicity phenotypes had the lowest false discovery rate (FDR), here defined as the average number of variants below a certain p-value (p-values in the range 0.00001–0.005 were tested) in the permutations divided by the number of true variants below the same p-value.

B2: Model based on cohort data with the addition of pathway over-representation analysis

Continuing on the SNV/INDEL association analysis, 1000 permutations with randomly and uniquely shuffled toxicity phenotype values were instead used to set different p-value thresholds for each toxicity phenotype to extract the top 50, 120, 210, and 400 SNVs from the SNV/INDEL association analysis.

To be able to extract both the most common and the most rare related variants from the SNV/INDEL association analysis, a gene/region-based association analysis of rare SNVs/INDELs was performed. For evaluating the association of common (MAF ≥0.01) and rare (MAF <0.01) genetic variants within a gene region (start and end of the gene region in GRCh38.103.gtf ± 6 base pairs) with the five TIPN phenotypes, the optimal sequence kernel association test in the R-package SKAT [5,6] was applied with default settings, using equal weighing of variants and age at survey (years), taxane type (docetaxel, paclitaxel, or both), docetaxel dose (mg/m^2), paclitaxel dose (mg/m^2), time since treatment (months), body mass index (BMI) at survey (kg/m^2), and diabetes (yes or no) as covariates. Genes containing only one genetic variant were excluded from this analysis.

ConsensusPathDB-human (CPDB) Release 35 (05.06.2021) [7](7) was used for enrichment analysis of genes represented by pertinent SNVs or genes from the SNV/INDEL and gene/region-based association analyses above. Over-representation analysis was done using pathway-based sets as defined by the pathway databases Wikipathways, Biocarta, Kegg, Reactome, and Pharmgkb using a

minimum overlap with the gene input list of two genes and a p-value cut-off of 0.5. For each extracted variant from the SNV/INDEL association analysis, the lowest p-value obtained from the over-representation analysis correlated to the variant were found and used in variant selection into the modelling.

C1: Combination of model A1 and B2

Using the previous meta-analysis of variants associated with taxane-induced neuropathy [1] used in the earlier A1 model, the most significant variants were extracted using the collected p-values in the study and a p-value threshold at 0.05 was set. These were then added to the previous B2 model of variants from the SNV/INDEL and gene/region-based association analyses with a selection using the over-representation analysis in CPDB. The p-value thresholds, based on analysis in CPDB, were adjusted to include significant SNVs from the meta-analysis in the previous study.

C2: Development of C1 using Variable Importance

To optimize the modelling even further, the last model incorporated the variable of importance into consideration during its building. The importance of each variable was collected from the building of the previous C1 model and the R function glm in the R package stats (version 3.6.2). By selecting the variables with the highest variable importance to the model, the number of variables included in the model could be minimized and could help find the most optimal result from the included variables.

References

1 Guijosa, A. *et al.* Pharmacogenetics of taxane-induced neurotoxicity in breast cancer: Systematic review and meta-analysis. *Clin Transl Sci* **15**, 2403-2436 (2022). https://doi.org:10.1111/cts.13370

2 Oshiro, C. *et al.* Taxane pathway. *Pharmacogenet Genomics* **19**, 979-983 (2009). https://doi.org:10.1097/FPC.0b013e3283335277

3 Kanehisa, M. & Goto, S. KEGG: kyoto encyclopedia of genes and genomes. *Nucleic Acids Res* **28**, 27-30 (2000). https://doi.org:10.1093/nar/28.1.27

4 Purcell, S. *et al.* PLINK: a tool set for whole-genome association and population-based linkage analyses. *Am J Hum Genet* **81**, 559-575 (2007). https://doi.org:10.1086/519795

5 Ionita-Laza, I., Lee, S., Makarov, V., Buxbaum, J. D. & Lin, X. Sequence kernel association tests for the combined effect of rare and common variants. *Am J Hum Genet* **92**, 841-853 (2013). https://doi.org:10.1016/j.ajhg.2013.04.015

6 Lee, S., Wu, M. C. & Lin, X. Optimal tests for rare variant effects in sequencing association studies. *Biostatistics* **13**, 762-775 (2012). https://doi.org:10.1093/biostatistics/kxs014

7 Herwig, R., Hardt, C., Lienhard, M. & Kamburov, A. Analyzing and interpreting genome data at the network level with ConsensusPathDB. *Nat Protoc* **11**, 1889-1907 (2016). https://doi.org:10.1038/nprot.2016.117

Supplementary Table S1
Genes and single nucleotide variants (SNV) included in the prediction models: A1, based on meta-analysis[1]; and C2 on final prediction models. The frequencies in training and test cohorts are divided into the frequencies of wildtype/wildtype (0), wildtype/variant (1), variant/variant (2). The allele frequency for all (being the training and test cohort combined) shows the variant frequency where a heterozygote trait has half of the weight of a homozygote trait.

GeneName	SNV	A1 *Numbness in feet*	C2 *Numbness in feet*	C2 *Tingling in feet*	Frequency in Training cohort (0;1;2)[1] [%]	Frequency in Test cohort (0;1;2) [%]	Allele Frequency in all [%]
**ABCA1**	**rs2230805**			1	59.5;35;5.5	67;32;1	21.3
**ABCA1**	**rs2230806**			1	54.4;38.4;7.2	61;38;1	24.4
**ABCB1**	**rs1045642**	1			26.6;47.3;26.2	22;57;21	49.7
**ABCB1**	**rs1128503**	1			39.7;44.3;16	30;53;17	39.7
**ABCB1**	**rs2229109**	1			86.5;13.1;0.4	88;12;0	6.7
**ABCB1**	**rs9282564**	1			79.7;17.7;2.5	79;19;2	11.5
**ABCC2**	**rs17222723**	1			93.7;5.9;0.4	91;9;0	3.7
**ABCC2**	**rs8187710**	1	1		93.2;6.3;0.4	91;9;0	3.9
**AC011483.1; KLK7**	**rs2659067**			1	84.4;15.2;0.4	89;11;0	7.3
**AC022335.1; SLCO1B1**	**rs11045819**	1			71.3;25.3;3.4	72;25;3	15.9
**AC022335.1; SLCO1B1**	**rs2306283**	1			38.4;45.1;16.5	39;42;19	39.3
**ADAMTS18**	**rs8059275**		1		66.2;32.9;0.8	78;20;2	15.7
**ADAMTS20**	**rs11182088**		1	1	43;46.4;10.5	37;53;10	34.6
**ADAMTS7**	**rs543268667**			1	97.5;2.5;0	97;3;0	1.3
**ADAMTS8**	**rs11222085**			1	66.2;31.2;2.5	62;35;3	18.9
**AL139260.1; AL139260.3; GJA9**	**rs874243**			1	56.5;32.5;11	47;48;5	27.7
**AL391628.1; PSEN2**	**rs6759**			1	27.8;50.2;21.9	31;49;20	46.3
**ALKBH7**	**rs7540**			1	80.2;19.4;0.4	81;18;1	10.1
**AMIGO2**	**rs2269828**			1	49.4;40.9;9.7	59;33;8	28.5
**AOC1**	**rs10156191**			1	50.6;38.8;10.5	50;45;5	29.2
**ATP6V0A2**	**rs17883456**		1	1	89.5;10.5;0	94;6;0	4.6
**ATP6V0D2; PSKH2**	**rs6998760**		1	1	41.8;39.7;18.6	37;56;7	37.4
**B3GNTL1**	**rs7225887**		1		58.6;37.1;4.2	54;41;5	23.6
**CABLES1**	**rs139352344**		1		65.8;29.1;5.1	65;31;4	19.5
**CALD1; AC083870.1**	**rs6973420**		1		28.7;47.7;23.6	27;51;22	47.4
**CARD11**	**rs61731201**			1	91.1;8.4;0.4	92;7;1	4.6
**CCDC88C**	**rs45437097**		1	1	56.5;38;5.5	61;35;4	23.5
**CCL11**	**rs1129844**			1	66.7;30.4;3	66;32;2	18.1
**CD40LG**	**rs1126535**			1	63.7;33.3;3	61;34;5	20.4
**CDH1**	**rs1801552**		1		40.5;44.3;15.2	39;53;8	36.5
**CHSY1**	**rs28364839**			1	54.9;39.2;5.9	68;29;3	23.1
**CHSY1**	**rs7175303**			1	65.8;32.5;1.7	78;22;0	15.9
**COL25A1**	**rs17531474**			1	73.8;24.9;1.3	73;25;2	14
**CSPG5**	**rs3732530**			1	47.7;42.6;9.7	42;41;17	33
**CST4**	**rs112139500**		1		75.9;22.8;1.3	78;21;1	12.3

**CYP1B1**	**rs1056836**	1			31.2;51.5;17.3	30;41;29	45
**CYP2C8**	**rs10509681**	1	1		75.5;23.6;0.8	80;19;1	12
**CYP2C8**	**rs1058930**	1		1	90.3;9.7;0	87;12;1	5.5
**CYP2C8**	**rs41286886**	1			96.6;3.4;0	98;2;0	1.5
**CYP2S1**	**rs57266494**			1	93.7;6.3;0	93;7;0	3.3
**DAZAP2**	**rs1049467**			1	59.1;36.3;4.6	52;38;10	24.6
**DHRS3**	**rs11540058**			1	62.9;35;2.1	69;29;2	18.7
**DHRS4AS1; DHRS4L2; AL136419.1**	**rs2273946**			1	44.7;40.9;14.3	48;36;16	34.5
**DLEC1; ACAA1**	**rs12630114**			1	44.3;43.5;12.2	49;34;17	33.9
**EGFR**	**rs2227984**		1		43;46.8;10.1	44;47;9	33.2
**EPHA5**	**rs33932471**	1			87.8;12.2;0	90;9;1	5.9
**EPHA5**	**rs36050417**	1			94.9;4.6;0.4	96;4;0	2.5
**EPHA5**	**rs7349683**	1	1	1	38;48.9;13.1	49;42;9	35.3
**EPHA6**	**rs4857276**	1			97.9;2.1;0	97;3;0	1.2
**ERCC2**	**rs13181**	1			35.9;49.8;14.3	37;43;20	39.9
**FABP2**	**rs1799883**		1		47.7;44.3;8	47;39;14	31.2
**GFRA4**	**rs2853208**			1	26.6;51.1;22.4	26;52;22	48
**GSTP1**	**rs1138272**	1	1		86.1;13.1;0.8	81;19;0	8
**GSTP1**	**rs1695**	1			51.9;40.9;7.2	39;47;14	30.6
**HGS**	**rs34384005**		1		88.2;10.5;1.3	87;13;0	6.5
**HLA-DRA**	**rs3135391**			1	73.4;24.9;1.7	74;25;1	14
**HSPG2**	**rs2228347**			1	43;47.3;9.7	48;37;15	33.4
**IFT140**	**rs2076436**		1		63.7;31.6;4.6	61;32;7	21.2
**IRS2**	**rs9583424**			1	49.8;43.5;6.8	50;44;6	28.3
**ITPR3**	**rs2229637**		1		52.3;40.1;7.6	54;40;6	27.1
**KAT6A**	**rs13748**			1	50.6;43.5;5.9	60;36;4	25.9
**KCNK4; KCNK4-TEX40**	**rs953778**		1		73.4;24.1;2.5	74;24;2	14.4
**KMT2C**	**rs74483926**			1	90.7;9.3;0	97;3;0	3.7
**LAMA1**	**rs617206**			1	31.6;45.6;22.8	28;50;22	46
**LAMA1**	**rs625106**			1	33.3;48.1;18.6	34;45;21	42.9
**LAMA2**	**rs1027199**			1	60.8;34.6;4.6	63;34;3	21.4
**LAMA2**	**rs3816665**			1	79.7;19;1.3	79;20;1	10.8
**LAMC3**	**rs117361076**			1	94.9;5.1;0	92;8;0	3
**LAMTOR5-AS1; LAMTOR5**	**rs6698159**		1		77.2;20.7;2.1	69;30;1	13.5
**LBR**	**rs1056608**		1		52.7;37.6;9.7	53;41;6	27.9
**LDLRAD2; HSPG2**	**rs3736360**			1	66.7;29.5;3.8	63;30;7	19.5
**MBL2**	**rs1800450**			1	69.6;29.1;1.3	71;29;0	15.4
**MC1R; AC092143.1; AC092143.2**	**rs2228478**			1	78.5;21.1;0.4	86;14;0	9.8
**MMP17**	**rs4964883**			1	94.9;5.1;0	95;4;1	2.7
**MUC4**	**rs74547899**		1		91.6;8;0.4	96;4;0	3.7
**NOTCH4**	**rs1044506**			1	72.6;25.7;1.7	69;31;0	14.8
**NR1H3**	**rs2279238**		1	1	70.5;26.2;3.4	73;26;1	15.8
**PATJ**	**rs41289430**			1	96.2;3.8;0	99;1;0	1.5
**PKHD1**	**rs765525**		1		41.4;45.1;13.5	47;42;11	34.9
**PLIN4**	**rs7260518**		1		82.3;17.3;0.4	82;17;1	9.2

**PLIN5**	**rs1062223**		1		65.4;28.7;5.9	65;28;7	20.4
**POLR1G; ERCC1**	**rs3212986**	1			57.4;38.4;4.2	47;50;3	24.8
**POU5F1**	**rs1265160**			1	68.4;29.5;2.1	66;29;5	17.7
**PREX2**	**rs3812458**		1		51.1;39.7;9.3	50;43;7	28.9
**PRKCI**	**rs56257047**			1	71.3;26.6;2.1	75;22;3	15
**PSMB9; TAP1**	**rs41551515**			1	91.1;8.9;0	90;9;1	4.8
**PSPN**	**rs2304198**			1	66.2;30;3.8	72;25;3	17.8
**RDH8**	**rs1644731**			1	27;47.7;25.3	32;50;18	47.3
**RELL2; FCHSD1**	**rs14251**		1		30.4;50.2;19.4	38;49;13	42.4
**RXRB**	**rs6531**		1		44.3;43.9;11.8	43;41;16	34.6
**SALL1**	**rs1965024**			1	46.4;41.4;12.2	41;42;17	34.4
**SBF2**	**rs12574508**	1			80.6;18.6;0.8	81;18;1	10.1
**SBF2; AC011092.2**	**rs7102464**	1			76.4;20.3;3.4	75;24;1	13.4
**SCN10A**	**rs57326399**			1	48.9;42.6;8.4	56;34;10	28.9
**SCN10A**	**rs7617919**		1		51.1;40.9;8	57;35;8	27.6
**SLC1A6**	**rs2229896**		1		68.8;28.7;2.5	73;23;4	16.5
**SLC38A9**	**rs4865614**		1		43.9;48.1;8	47;38;15	32.7
**SLCO1B1**	**rs34671512**	1			90.7;9.3;0	91;9;0	4.6
**SLCO1B1**	**rs4149056**	1			72.2;24.9;3	72;26;2	15.3
**SLCO1B3; SLCO1B3-SLCO1B7**	**rs4149117**	1			69.2;27.8;3	64;32;4	17.8
**SLCO1B3; SLCO1B3-SLCO1B7**	**rs60140950**	1			76.4;19.8;3.8	73;27;0	13.7
**SPTB**	**rs229592**			1	50.6;43;6.3	49;44;7	28.2
**STK10**	**rs2306961**		1		56.5;36.3;7.2	55;42;3	24.9
**UST**	**rs9498146**			1	93.2;6.3;0.4	92;8;0	3.7
**ZBTB33**	**rs201958171**		1		50.2;43.5;6.3	44;50;6	28.9

Genes marked in grey are discussed in the manuscript.

1 Guijosa, A. *et al.* Pharmacogenetics of taxane-induced neurotoxicity in breast cancer: Systematic review and meta-analysis. *Clin Transl Sci* **15**, 2403-2436 (2022). https://doi.org:10.1111/cts.13370

**Supplementary Table S2 Model description**							
**NUMBNESS MODEL A1**							
Deviance	Residuals:						
Min	1Q	Median	3Q	Max			
-2.1380	-0.7193	-0.4782	0.5358	2.5403			
Coefficients:							
	Estimate	Std. Error	z value	CI (2.5 %)	CI (97.5 %)	Pr(>|z|)	
(Intercept)	-0.684553	0.631278	-1.084	-1.942575e+00	0.5454869	0.27819	
rs1056836	-0.287042	0.268706	-1.068	-8.239420e-01	0.2347723	0.28541	
rs4857276	0.738584	1.089732	0.678	-1.564302e+00	2.8907453	0.49792	
rs7349683	-0.160315	0.286094	-0.560	-7.298536e-01	0.3974466	0.57524	
rs36050417	1.847944	0.763557	2.420	4.182849e-01	3.4102971	0.01551	*
rs33932471	0.043330	0.559679	0.077	-1.100439e+00	1.1126922	0.93829	
rs1045642	0.264452	0.335602	0.788	-3.962339e-01	0.9270336	0.43070	
rs1128503	-0.278175	0.344302	-0.808	-9.627328e-01	0.3948529	0.41913	
rs2229109	0.294846	0.500949	0.589	-7.123602e-01	1.2764217	0.55615	
rs9282564	-0.425451	0.468803	-0.908	-1.397742e+00	0.4537719	0.36413	
rs10509681	0.241640	0.392791	0.615	-5.462444e-01	1.0032283	0.53843	
rs1058930	0.053003	0.591974	0.090	-1.167971e+00	1.1807134	0.92866	
rs41286886	1.059193	0.887399	1.194	-7.791556e-01	2.7881223	0.23264	
rs17222723	13.350390	882.744228	0.015	-1.654531e+02	NA	0.98793	
rs8187710	-13.084804	882.743945	-0.015	NA	166.0874118	0.98817	
rs12574508	0.072383	0.426100	0.170	-7.991258e-01	0.8830777	0.86511	
rs7102464	-0.449207	0.371457	-1.209	-1.214795e+00	0.2544138	0.22654	
rs1695	-0.436742	0.352602	-1.239	-1.152276e+00	0.2384737	0.21548	
rs1138272	-0.516615	0.598196	-0.864	-1.771485e+00	0.6097407	0.38780	
rs4149117	0.230896	0.372619	0.620	-5.102081e-01	0.9626290	0.53548	
rs60140950	-0.269030	0.506674	-0.531	-1.304860e+00	0.6986414	0.59544	
rs2306283	-0.483403	0.468177	-1.033	-1.446343e+00	0.3946329	0.30183	
rs11045819	-0.037065	0.675481	-0.055	-1.349486e+00	1.3134511	0.95624	
rs4149056	0.316233	0.496237	0.637	-6.570087e-01	1.3036473	0.52395	
rs34671512	1.042613	0.768833	1.356	-4.547084e-01	2.5846269	0.17507	
rs13181	0.008674	0.270527	0.032	-5.260314e-01	0.5397481	0.97442	
rs3212986	0.235325	0.307591	0.765	-3.717960e-01	0.8406997	0.44424	
Age	0.359122	0.190778	1.882	-5.746767e-03	0.7454834	0.05978	.
Diabetes	0.541693	0.170266	3.181	2.206500e-01	0.8984277	0.00147	**
BMI	0.265004	0.175044	1.514	-8.243310e-02	0.6089247	0.13005	
Taxane_type	0.493912	0.181512	2.721	1.431281e-01	0.8585474	0.00651	**

Signif. codes: 0 ‘***’ 0.001 ‘**’ 0.01 ‘*’ 0.05 ‘.’ 0.1 ‘ ’ 1

(Dispersion parameter for binomial family taken to be 1)

Null deviance: 269.13 on 234 degrees of freedom
Residual deviance: 220.76 on 204 degrees of freedom
AIC: 282.76

Number of Fisher Scoring iterations: 13

**Prediction accuracy**		**TRAIN**	**TEST**
	F1 score	0,5840708	0,342857143
	Precision	0,54098361	0,24
	Recall	0,63461538	0,6
	Accuarcy	0,8	0,77

Confusion matrix

TRAIN:

	FALSE	TRUE
FALSE	155	28
TRUE	19	33

TEST:

	FALSE	TRUE
FALSE	71	19
TRUE	4	6

**NUMBNESS MODEL C2**							
Deviance Residuals:							
Min	1Q	Median	3Q	Max			
-2.7031	-0.5863	-0.2796	0.2364	2.5758			
Coefficients:							
	Estimate	Std. Error	z value	CI (2.5 %)	CI (97.5 %)	Pr(>|z|)	
(Intercept)	-2.98340	1.05089	-2.839	-5.115897533	-0.96656016	0.004527	**
rs7349683	-0.54781	0.35445	-1.545	-1.268906294	0.13229901	0.122226	
rs10509681	0.63922	0.44986	1.421	-0.249184485	1.53034362	0.155341	
rs8187710	-1.17159	0.85760	-1.366	-2.929722116	0.45282360	0.171897	
rs1138272	-1.59790	0.69023	-2.315	-3.069317412	-0.34284738	0.020610	*
rs74547899	-1.72325	1.00542	-1.714	-3.992004079	0.02170903	0.086535	.
rs2229637	-0.71218	0.38779	-1.837	-1.517759175	0.01331262	0.066281	.
rs14251	0.52300	0.29741	1.759	-0.050388796	1.12385325	0.078656	.
rs6531	0.47426	0.32559	1.457	-0.152249607	1.13307967	0.145222	
rs2279238	-0.30238	0.41987	-0.720	-1.149979404	0.50797067	0.471415	
rs7617919	0.16671	0.35513	0.469	-0.539501876	0.86356576	0.638767	
rs6973420	0.31892	0.32433	0.983	-0.313990236	0.96668394	0.325455	
rs953778	-0.45552	0.46730	-0.975	-1.421282366	0.42361921	0.329660	
rs1056608	-0.28134	0.35918	-0.783	-1.016369663	0.40224218	0.433458	
rs6698159	-0.35884	0.45049	-0.797	-1.287016288	0.49869267	0.425703	
rs4865614	-0.61779	0.35736	-1.729	-1.343805497	0.06680324	0.083854	.
rs2227984	0.05183	0.35410	0.146	-0.658836717	0.74049367	0.883634	
rs3812458	0.66242	0.34142	1.940	0.001928627	1.35191144	0.052358	.

rs11182088	0.27863	0.31528	0.884	-0.340256619	0.90420191	0.376828	
rs8059275	0.98412	0.46764	2.104	0.083729039	1.93243652	0.035340	*
rs7225887	-0.47048	0.37873	-1.242	-1.242775716	0.25460095	0.214136	
rs2306961	0.75661	0.33648	2.249	0.107784773	1.43729833	0.024539	*
rs765525	0.13284	0.31072	0.428	-0.480890932	0.74667275	0.669008	
rs45437097	-0.31460	0.35523	-0.886	-1.026964015	0.37468447	0.375827	
rs2076436	0.17115	0.36412	0.470	-0.549219814	0.89044564	0.638321	
rs112139500	0.73322	0.48228	1.520	-0.225358808	1.68725613	0.128430	
rs1799883	0.20698	0.34677	0.597	-0.486097716	0.88330814	0.550581	
rs7260518	0.27870	0.52878	0.527	-0.773850657	1.31303116	0.598143	
rs1062223	0.39654	0.35306	1.123	-0.301783897	1.09245932	0.261369	
rs1801552	-0.50645	0.30573	-1.657	-1.123741566	0.08223492	0.097611	.
rs34384005	0.31341	0.54273	0.577	-0.770665513	1.38162252	0.563619	
rs139352344	-0.37426	0.39312	-0.952	-1.177275323	0.37621471	0.341077	
rs201958171	1.51884	0.41284	3.679	0.746848877	2.37602535	0.000234	***
rs6998760	0.63179	0.31814	1.986	0.024227655	1.28072617	0.047043	*
rs17883456	-2.99014	1.11633	-2.679	-5.579760670	-1.07291832	0.007394	**
rs2229896	-0.60523	0.40604	-1.491	-1.435196655	0.16940834	0.136070	
Age	0.74629	0.23793	3.137	0.300567058	1.23920757	0.001709	**
Diabetes	0.68804	0.22072	3.117	0.280069771	1.15736382	0.001825	**
BMI	0.40387	0.21594	1.870	-0.018251924	0.83580948	0.061445	.
Taxane_type	0.64342	0.22628	2.844	0.215164385	1.10899571	0.004462	**

Signif. codes: 0 ‘***’ 0.001 ‘**’ 0.01 ‘*’ 0.05 ‘.’ 0.1 ‘ ’ 1

(Dispersion parameter for binomial family taken to be 1)

Null deviance: 269.13 on 234 degrees of freedom
Residual deviance: 175.85 on 195 degrees of freedom
AIC: 255.85

Number of Fisher Scoring iterations: 6

**Prediction accuracy**		**TRAIN**	**TEST**
	F1 score	0,63551402	0,476190476
	Precision	0,55737705	0,4
	Recall	0,73913043	0,588235294
	Accuarcy	0,83404255	0,78

Confusion matrix

TRAIN:

	FALSE	TRUE
FALSE	162	27
TRUE	12	34

TEST:

	FALSE	TRUE
FALSE	68	15
TRUE	7	10

**TINGLING MODEL C2**							
Deviance Residuals:							
Min	1Q	Median	3Q	Max			
-1.5616	-0.6773	-0.2532	0.1756	2.7277			
Coefficients:							
	Estimate	Std. Error	z value	CI (2.5 %)	CI (97.5 %)	Pr(>|z|)	
(Intercept)	-3.873985	1.270079	-3.050	-6.52379605	-1.50349904	0.002287	**
rs7349683	-0.918266	0.404903	-2.268	-1.75703348	-0.15893044	0.023337	*
rs1058930	2.734319	0.868181	3.149	1.09378202	4.52749095	0.001636	**
rs57266494	-1.181028	1.023443	-1.154	-3.39184352	0.69741159	0.248510	
rs1644731	-0.120743	0.319337	-0.378	-0.75391667	0.50859071	0.705351	
rs11540058	-1.054189	0.490288	-2.150	-2.05887671	-0.12223939	0.031544	*
rs41551515	-1.976468	1.185948	-1.667	-4.58193209	0.10283573	0.095600	.
rs3135391	-1.849694	0.945763	-1.956	-3.77114890	-0.02796714	0.050492	.
rs117361076	-3.157851	1.360395	-2.321	-6.14907414	-0.70996085	0.020272	*
rs1027199	1.117672	0.457582	2.443	0.23396719	2.04241698	0.014583	*
rs617206	-0.662242	0.326622	-2.028	-1.32679925	-0.03877397	0.042607	*
rs625106	-0.430826	0.332673	-1.295	-1.10031800	0.21284714	0.195305	
rs3816665	-0.853304	0.593207	-1.438	-2.08794563	0.26236783	0.150304	
rs3736360	0.481680	0.570918	0.844	-0.61640309	1.63982095	0.398840	
rs2230392	-1.283069	0.655908	-1.956	-2.67008635	-0.07255265	0.050445	.
rs2228347	-0.016115	0.493951	-0.033	-1.01233798	0.93837109	0.973974	
rs56180838	0.203140	0.583468	0.348	-0.97103198	1.33721081	0.727720	
rs229592	0.522121	0.381195	1.370	-0.21906204	1.28596338	0.170782	
rs2230805	-1.278968	0.694527	-1.841	-2.67485168	0.08200591	0.065549	.
rs2230806	1.687223	0.672164	2.510	0.38038780	3.05004357	0.012068	*
rs10156191	0.322283	0.329231	0.979	-0.32358756	0.97741058	0.327630	
rs12630114	0.186640	0.314326	0.594	-0.43653102	0.80510392	0.552661	
rs11182088	0.282485	0.345430	0.818	-0.39309774	0.97323336	0.413484	
rs11222085	0.151821	0.441843	0.344	-0.72480897	1.02173292	0.731140	
rs543268667	0.884340	1.338756	0.661	-1.89876322	3.45222276	0.508889	
rs2279238	-0.981639	0.483785	-2.029	-1.98059387	-0.07106382	0.042450	*
rs9583424	0.437452	0.367502	1.190	-0.28101953	1.17177828	0.233913	
rs1265160	1.733552	0.500111	3.466	0.79764481	2.77353481	0.000528	***
rs2269828	0.825323	0.394670	2.091	0.07465190	1.63672106	0.036513	*
rs1126535	0.835774	0.415519	2.011	0.03502906	1.67692120	0.044284	*
rs56257047	0.471675	0.423478	1.114	-0.35673550	1.31665922	0.265360	
rs1044506	1.733627	0.946044	1.833	-0.10024510	3.64862857	0.066877	.
rs17883456	-0.895892	0.841475	-1.065	-2.64403710	0.69683749	0.287026	
rs41289430	1.953085	1.242979	1.571	-0.49565205	4.42228673	0.116115	
rs6998760	0.498758	0.349501	1.427	-0.17883962	1.20289785	0.153563	
rs1129844	-0.723990	0.445599	-1.625	-1.63401308	0.12573717	0.104214	
rs45437097	-0.549497	0.416635	-1.319	-1.38668960	0.26065797	0.187204	
rs2853208	0.236293	0.333964	0.708	-0.41352770	0.90567341	0.479230	
rs2304198	-0.549231	0.644615	-0.852	-1.90632302	0.66067393	0.394197	
rs7540	1.174296	0.788101	1.490	-0.33046926	2.79639175	0.136216	

rs7175303	-0.004059	0.757925	-0.005	-1.46265523	1.54914690	0.995727	
rs28364839	0.389145	0.669732	0.581	-0.99183528	1.67374356	0.561209	
rs874243	0.985423	0.385323	2.557	0.25021952	1.77055943	0.010546	*
rs13748	0.209482	0.395737	0.529	-0.57034484	0.99406803	0.596565	
rs74483926	2.225991	0.734715	3.030	0.81528237	3.72702293	0.002448	**
rs2228478	1.120446	0.551126	2.033	0.05301336	2.23199515	0.042051	*
rs1800450	-1.040416	0.515443	-2.018	-2.10322065	-0.06708617	0.043540	*
rs2659067	-1.363701	0.795626	-1.714	-3.03533870	0.11259307	0.086529	.
rs4964883	-0.731880	1.282061	-0.571	-3.73771761	1.52233727	0.568093	
rs17531474	1.323747	0.520223	2.545	0.32512711	2.38508582	0.010941	*
rs6759	0.303480	0.351515	0.863	-0.37631468	1.01331182	0.387945	
rs61731201	-0.588633	0.695197	-0.847	-2.01447659	0.74711865	0.397154	
rs1049467	0.006814	0.404965	0.017	-0.80177542	0.79833778	0.986576	
rs3732530	-0.921967	0.398564	-2.313	-1.74213645	-0.16768199	0.020711	*
rs9498146	0.707518	0.943759	0.750	-1.25908200	2.53073691	0.453447	
rs1965024	0.733523	0.344286	2.131	0.07405361	1.43541926	0.033125	*
Age	0.628521	0.255939	2.456	0.14688997	1.15852416	0.014059	*
Diabetes	0.888417	0.239688	3.707	0.44605424	1.39507297	0.000210	***
BMI	-0.026209	0.237969	-0.110	-0.49690057	0.44303548	0.912301	
Taxane_type	0.249040	0.223899	1.112	-0.19157080	0.69416936	0.266015	

Signif. codes: 0 ‘***’ 0.001 ‘**’ 0.01 ‘*’ 0.05 ‘.’ 0.1 ‘ ’ 1

(Dispersion parameter for binomial family taken to be 1)

Null deviance: 273.23 on 234 degrees of freedom
Residual deviance: 180.47 on 175 degrees of freedom
AIC: 300.47

Number of Fisher Scoring iterations: 6

**Prediction accuracy**	**TRAIN**	**TEST**
F1 score	0,62264151	0,095238095
Precision	0,52380952	0,05
Recall	0,76744186	1,0
Accuarcy	0,82978723	0,81

Confusion matrix

TRAIN:

	FALSE	TRUE
FALSE	162	30
TRUE	10	33

TEST:

	FALSE	TRUE
FALSE	80	19
TRUE	0	1

Supplementary Table S3 Model performance

A1: Variants based on previous meta-analysis

Symptoms	SNVs (genes)	Cutoff	Set	AUC (%)	(%)	(%)	(%)
Numbness in feet	26 (18)	0,39621	Train	78,74	80,43	55,74	89,08
			Test	**67,09**	**67,09**	**67,09**	**67,09**
Tingling in feet	26 (18)	0,49661	Train	75,42	79,57	33,33	96,51
			Test	*56,31*	*75,00*	*25,00*	*87,50*
Cramps in feet	26 (18)	0,46297	Train	72,06	75,32	33,33	93,87
			Test	*51,48*	*68,00*	*9,52*	*83,54*
Difficulty opening a jar	26 (18)	0,41136	Train	76,00	80,59	48,28	91,06
			Test	*55,71*	*63,00*	*20,00*	*81,43*
Difficulty climbing	26 (18)	0,39425	Train	84,04	91,98	42,86	98,56
			Test	*32,80*	*78,00*	*0,00*	*89,66*
Summa SNVs and	26 (18)	-	-	-	-	-	-

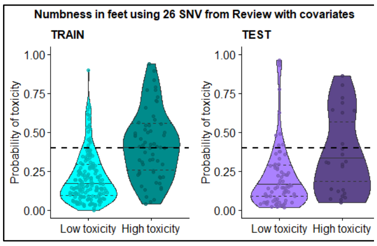

A2: Model based on predefined and previously known SNVs and genes

Symptoms	SNVs (genes)	Cutoff	Set	AUC (%)	Accuracy (%)	Sensitivity (%)	Specificity (%)
Numbness in feet	54 (13)	0,44140	Train	85,22	82,98	65,57	89,08
			Test	*58,08*	*68,00*	*12,00*	*86,67*
Tingling in feet	54 (13)	0,54063	Train	82,94	79,15	42,86	92,44
			Test	*52,94*	*76,00*	*25,00*	*88,75*
Cramps in feet	54 (13)	0,51282	Train	85,11	81,70	56,94	92,64
			Test	*56,90*	*70,00*	*19,05*	*83,54*
Difficulty opening a jar	54 (13)	0,54110	Train	86,67	83,54	48,28	94,97
			Test	*47,33*	*59,00*	*16,67*	*77,14*
Difficulty climbing	54 (13)	0,38721	Train	93,80	94,09	67,86	97,61
			Test	*53,36*	*75,00*	*15,38*	*83,91*
Summa SNVs and	54 (13)	-	-	-	-	-	-

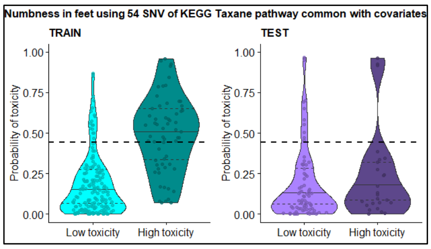

B1: Model based on cohort data using SNV/INDEL association analysis

Symptoms	FDR perm threshold	SNVs (genes)	Cutoff	Set	AUC (%)	Accuracy (%)	Sensitivity (%)	Specificity (%)
Numbness in feet	(71)	48 (52)	0,46722	Train	85,86	81,70	54,10	91,38
				Test	*59,63*	*67,00*	*36,00*	*77,33*
Tingling in feet	(79)	63 (74)	0,45897	Train	82,18	82,13	50,79	93,60
				Test	*56,62*	*66,00*	*35,00*	*73,75*
Cramps in feet	0.001125	47 (50)	0,55944	Train	78,15	76,60	34,72	95,09
				Test	*40,14*	*64,00*	*4,76*	*79,75*
Difficulty opening a jar	0.0005 (33)	27 (34)	0,45775	Train	72,55	78,90	27,59	95,53
				Test	*55,62*	*67,00*	*20,00*	*87,14*
Difficulty climbing	0.0005 (46)	40 (45)	0,50806	Train	88,52	91,14	42,86	97,61
				Test	*50,40*	*81,00*	*23,08*	*89,66*
Summa SNVs and	(282)	212 (234)	-	-	-	-	-	-

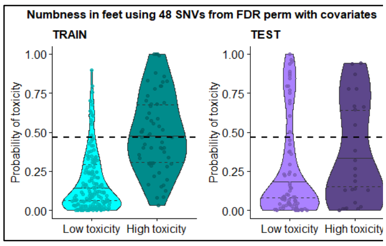

B2: Model based on cohort data with the addition of pathway over-representation analysis

Symptoms	FDR perm threshold (SNVs)	P (CPDB)	SNVs (genes)	Cutoff	Set	AUC (%)	Accuracy (%)	Sensitivity (%)	Specificity (%)
Numbness in feet	0,006 (398)	0,005	82 (74)	0,51626	Train	93,70	88,94	73,77	94,25
					Test	**68,43**	**69,00**	**56,00**	**73,33**
Tingling in feet	0,006 (408)	0,019	101 (111)	0,64339	Train	95,97	92,34	74,60	98,84
					Test	*47,72*	*65,00*	*30,00*	*73,75*
Cramps in feet	0,007 (213)	0,200	96 (101)	0,59641	Train	96,20	91,06	77,78	96,93
					Test	*39,90*	*53,00*	*28,57*	*59,49*
Difficulty opening a jar	0,007 (213)	0,160	90 (97)	0,51437	Train	94,94	90,30	72,41	96,09
					Test	*38,57*	*55,00*	*23,33*	*68,57*
Difficulty climbing	0,006 (213)	0,075	60 (65)	0,64974	Train	98,67	98,73	89,29	100,00
					Test	*50,31*	*76,00*	*23,08*	*83,91*
Summa SNVs and	(1119)	-	266 (265)	-	-	-	-	-	-

C1: Combination of model A1 and B2

Symptoms	FDR perm threshold	p (CPDB)	Meta-analysis	SNVs (genes)	Cutoff	Set	AUC (%)	Accuracy (%)	Sensitivity (%)	Specificity (%)
Numbness in feet	0,006 (398)	0,0025	0,05 (8)	86 (77)	0,45648	Train	97,41	93,62	86,89	95,98
						Test	**71,15**	**67,00**	**56,00**	**70,67**
Tingling in feet	0,006 (408)	0,0190	0,05 (7)	108 (116)	0,50406	Train	96,49	93,62	87,30	95,93
						Test	*52,34*	*59,00*	*25,00*	*67,50*
Cramps in feet	0,007 (213)	0,1300	0,05 (8)	95 (99)	0,47864	Train	96,69	92,34	87,50	94,48
						Test	*40,11*	*51,00*	*23,81*	*58,23*
Difficulty opening a jar	0,007 (213)	0,1100	0,05 (8)	88 (90)	0,48820	Train	92,57	87,76	70,69	93,30
						Test	*45,48*	*60,00*	*26,67*	*74,29*
Difficulty climbing	0,006 (213)	0,0600	0,05 (8)	60 (63)	0,55089	Train	98,05	98,31	85,71	100,00
						Test	*40,85*	*74,00*	*7,69*	*83,91*
Summa SNVs and	(1119)	-	(8)	260 (259)	-	-	-	-	-	-

C2: Development of C1 using Variable Importance

Symptoms	FDR perm threshold	p (CPDB)	Meta-analysis	VarImp threshold	SNVs (genes)	Cutoff	Set	AUC (%)	Accuracy (%)	Sensitivity (%)	Specificity (%)
Numbness in feet	0,006 (398)	0,0025	0,05 (8)	1,67	35 (40)	0,54259	Train	88,87	83,4	55,74	93,1
							Test	**72,91**	**73**	**44**	**82,67**
Tingling in feet	0,006 (408)	0,0190	0,05 (7)	1,2	55 (60)	0,50883	Train	85,96	81,28	49,21	93,02
							Test	**60,88**	**72**	**40**	**80**
Cramps in feet	0,007 (213)	0,1300	0,05 (8)	1,2	50 (57)	0,448	Train	85,69	80,43	65,28	87,12
							Test	*42,98*	*52*	*28,57*	*58,23*
Difficulty opening a jar	0,007 (213)	0,1100	0,05 (8)	1,1	33 (42)	0,66991	Train	80,34	78,06	15,52	98,32
							Test	*51,71*	*67*	*13,33*	*90*
Difficulty climbing	0,006 (213)	0,0600	0,05 (8)	1	35 (38)	0,64398	Train	90,02	91,56	39,29	98,56
							Test	*42,88*	*75*	*0*	*86,21*
Summa SNVs and	(1119)	-	(8)	-	(158)	-	-	-	-	-	-

Supplementary Table S4 CPDB results

A) C2 Numbness 35 SNVs

p-value	q-value	pathway	source	external_id	members_input_overlap	members_input_overlap_ge	size	effective_size
2.01000839451e-07	3.3165138509	Posttranslational regulation of adherens junctio	PID	ajdiss_2pathw	CDH1; CABLES1; ZBTB33; EG	10009; 999; 9146; 91768; 1!	53	53
1.08639445685e-06	8.9627542690	PPAR signaling pathway - Homo sapiens (human)	KEGG	path:hsa0332	PLIN4; PLIN5; FABP2; RXRB;	729359; 440503; 10062; 62	76	74
6.18790801347e-05	0.0020420096	NR1H2 & NR1H3 regulate gene expression linkec	Reactome	R-HSA-90315	RXRB; NR1H3	10062; 6257	5	5
6.18790801347e-05	0.0020420096	NR1H2 & NR1H3 regulate gene expression linkec	Reactome	R-HSA-96329	RXRB; NR1H3	10062; 6257	5	5
6.18790801347e-05	0.0020420096	NR1H2 & NR1H3 regulate gene expression to lim	Reactome	R-HSA-90315	RXRB; NR1H3	10062; 6257	5	5
8.40058333209e-05	0.0023101604	Amino acids regulate mTORC1	Reactome	R-HSA-96392	ATP6V0D2; SLC38A9; LAMT	245972; 10542; 153129	34	34
0.00010133742941	0.0023886679	Signal Transduction	Reactome	R-HSA-16258	IFT140; LBR; PREX2; PKHD1	23545; 6793; 80243; 1956; !	####	2431
0.00012953382971	0.0026716352	Arachidonate Epoxygenase - Epoxide Hydrolase	Wikipathw	WP678	CYP2C8; GSTP1	1558; 2950	7	7
0.00019382418952	0.0030436139	O-linked glycosylation	Reactome	R-HSA-51731	ADAMTS20; ADAMTS18; B3(	170692; 146712; 80070; 45	115	115
0.00019545215085	0.0030436139	a6b1 and a6b4 Integrin signaling	PID	a6b1_a6b4_i	CDH1; EGFR; RXRB	999; 1956; 6257	45	45
0.00022135374312	0.0030436139	NR1H2 & NR1H3 regulate gene expression linkec	Reactome	R-HSA-90295	RXRB; NR1H3	10062; 6257	9	9
0.00022135374312	0.0030436139	NR1H2 & NR1H3 regulate gene expression to cor	Reactome	R-HSA-96234	RXRB; NR1H3	10062; 6257	9	9
0.00045126599905	0.0057276069	Intracellular signaling by second messengers	Reactome	R-HSA-90069	PREX2; SLC38A9; LAMTOR5	1956; 153129; 3710; 10542	259	259
0.00066340911609	0.0070823172	PPAR signaling pathway	Wikipathw	WP3942	FABP2; RXRB; NR1H3	10062; 2169; 6257	68	68
0.00072212220229	0.0070823172	Epithelial cell signaling in Helicobacter pylori inf	KEGG	path:hsa0512	ATP6V0D2; EGFR; ATP6V0A	23545; 1956; 245972	70	70
0.00072969328876	0.0070823172	Mycophenolic Acid Metabolism Pathway	SMPDB	SMP00652	CYP2C8; ABCC2	1244; 1558	16	16
0.00072969328876	0.0070823172	Codeine and Morphine Metabolism	Wikipathw	WP1604	CYP2C8; ABCC2	1558; 1244	16	16
0.00093089167775	0.0085331737	Muscle contraction	Reactome	R-HSA-39701	KCNK4; ITPR3; CALD1; SCN1	50801; 6336; 3710; 800	174	174
0.00098982608456	0.0085377353	Synaptic vesicle cycle - Homo sapiens (human)	KEGG	path:hsa0472	ATP6V0D2; SLC1A6; ATP6V(	23545; 245972; 6511	78	78
0.00103487701637	0.0085377353	Listeria monocytogenes entry into host cells	Reactome	R-HSA-88763	CDH1; HGS	9146; 999	19	19
0.00126687794779	0.0099540410	E-cadherin signaling in keratinocytes	PID	ecadherin_ke	CDH1; EGFR	999; 1956	21	21
0.00139135847327	0.0104351885	mTORC1-mediated signalling	Reactome	R-HSA-16620	LAMTOR5; SLC38A9	10542; 153129	22	22
0.00152145549897	0.0109147894	Photodynamic therapy-induced NFE2L2 (NRF2) :	Wikipathw	WP3612	GSTP1; ABCC2	2950; 1244	23	23
0.00164528115314	0.0113113079	PTEN Regulation	Reactome	R-HSA-68070	PREX2; SLC38A9; LAMTOR5	153129; 80243; 10542	93	93
0.00194517071972	0.0123443526	RXR and RAR heterodimerization with other nuc	PID	rxr_vdr_path	RXRB; NR1H3	10062; 6257	26	26
0.00194517071972	0.0123443526	Insulin receptor recycling	Reactome	R-HSA-77387	ATP6V0D2; ATP6V0A2	245972; 23545	26	26
0.00209745936909	0.0125847867	Collecting duct acid secretion - Homo sapiens (h	KEGG	path:hsa0496	ATP6V0D2; ATP6V0A2	245972; 23545	27	27
0.00213560017839	0.0125847867	PIP3 activates AKT signaling	Reactome	R-HSA-12576	SLC38A9; EGFR; PREX2; LAN	80243; 1956; 153129; 1054	218	218
0.0023281439312	0.0128723897	Cardiac conduction	Reactome	R-HSA-55768	KCNK4; ITPR3; SCN10A	50801; 6336; 3710	105	105
0.00239183165747	0.0128723897	Parathyroid hormone synthesis, secretion and a	KEGG	path:hsa0492	ITPR3; EGFR; RXRB	6257; 3710; 1956	106	106
0.00241844898586	0.0128723897	Energy dependent regulation of mTOR by LKB1-A	Reactome	R-HSA-38097	LAMTOR5; SLC38A9	10542; 153129	29	29
0.0025870959378	0.0133397134	EGFR downregulation	Reactome	R-HSA-18297	EGFR; HGS	9146; 1956	30	30
0.00276114179967	0.0138057089	Transferrin endocytosis and recycling	Reactome	R-HSA-91797	ATP6V0D2; ATP6V0A2	245972; 23545	31	31
0.00294055980009	0.0142703637	Gastric Cancer Network 2	Wikipathw	WP2363	LBR; EGFR	3930; 1956	32	32
0.0035107803278	0.0160910765	Nuclear Receptors in Lipid Metabolism and Toxi	Wikipathw	WP299	ABCC2; NR1H3	10062; 1244	35	35
0.0035107803278	0.0160910765	ROS and RNS production in phagocytes	Reactome	R-HSA-12225	ATP6V0D2; ATP6V0A2	245972; 23545	35	35
0.00371142104519	0.0165509316	Methadone Action Pathway	SMPDB	SMP00408	CYP2C8; SCN10A	1558; 6336	36	36
0.00391730144915	0.0165731984	Thyroid cancer - Homo sapiens (human)	KEGG	path:hsa0521	CDH1; RXRB	999; 6257	37	37
0.00391730144915	0.0165731984	NR1H3 & NR1H2 regulate gene expression linkec	Reactome	R-HSA-90295	RXRB; NR1H3	10062; 6257	37	37
0.0041283953283	0.0170296307	Nuclear receptors	Wikipathw	WP170	RXRB; NR1H3	10062; 6257	38	38
0.00434467656359	0.0170683722	Internalization of ErbB1	PID	erbb1_intern	EGFR; HGS	9146; 1956	39	39
0.00434467656359	0.0170683722	O-glycosylation of TSR domain-containing prote	Reactome	R-HSA-51732	ADAMTS20; ADAMTS18	80070; 170692	39	39
0.00456611912806	0.0175211547	Bladder cancer	Wikipathw	WP2828	CDH1; EGFR	999; 1956	40	40
0.00479269708654	0.0175732226	Bladder cancer - Homo sapiens (human)	KEGG	path:hsa0521	CDH1; EGFR	999; 1956	41	41
0.00479269708654	0.0175732226	MTOR signalling	Reactome	R-HSA-16515	LAMTOR5; SLC38A9	10542; 153129	41	41
0.00502438459537	0.0180222490	Stabilization and expansion of the E-cadherin ad	PID	ecadherin_st	CDH1; EGFR	999; 1956	42	42
0.00576952560781	0.0198589152	Cellular response to starvation	Reactome	R-HSA-97110	ATP6V0D2; SLC38A9; LAMT	245972; 10542; 153129	145	145
0.00585126124055	0.0198589152	Signaling by Receptor Tyrosine Kinases	Reactome	R-HSA-90069	ITPR3; EGFR; HGS; ATP6V0A	23545; 3710; 1956; 245972	464	464
0.00600171644898	0.0198589152	Cholesterol metabolism (includes both Bloch an	Wikipathw	WP4718	LBR; NR1H3	10062; 3930	46	46
0.00622093406141	0.0198589152	Gastric cancer - Homo sapiens (human)	KEGG	path:hsa0522	CDH1; EGFR; RXRB	999; 6257; 1956	149	149
0.00622093406141	0.0198589152	RHOA GTPase cycle	Reactome	R-HSA-89806	STK10; LBR; PREX2	6793; 80243; 3930	149	149
0.00625856723873	0.0198589152	NR1H2 and NR1H3-mediated signaling	Reactome	R-HSA-90244	RXRB; NR1H3	10062; 6257	47	47
0.00645436332487	0.0199233662	Phagosome - Homo sapiens (human)	KEGG	path:hsa0414	ATP6V0D2; HGS; ATP6V0A2	245972; 9146; 23545	152	151
0.00652037442346	0.0199233662	Signaling by EGFR	Reactome	R-HSA-17792	EGFR; HGS	9146; 1956	48	48
0.00678711279246	0.0203613383	NHR	Signalink	None	RXRB; NR1H3	10062; 6257	49	49
0.00705875722439	0.0207981239	Vibrio cholerae infection - Homo sapiens (humar	KEGG	path:hsa0511	ATP6V0D2; ATP6V0A2	245972; 23545	50	50
0.00790287701853	0.0228767492	Nuclear Receptor transcription pathway	Reactome	R-HSA-38328	RXRB; NR1H3	10062; 6257	53	53
0.00807561818759	0.0229737413	Nuclear Receptors Meta-Pathway	Wikipathw	WP2882	GSTP1; EGFR; ABCC2; NR1H	10062; 2950; 1956; 1244	317	317
0.00909554645212	0.0250127527	Proximal tubule transport	Wikipathw	WP4917	ATP6V0D2; ABCC2	245972; 1244	57	57
0.00909554645212	0.0250127527	Iron uptake and transport	Reactome	R-HSA-91793	ATP6V0D2; ATP6V0A2	245972; 23545	57	57
0.00940554565028	0.0254412300	Endometrial cancer - Homo sapiens (human)	KEGG	path:hsa0521	CDH1; EGFR	1956; 999	58	58

B) C2 Tingling 55 SNVs

p-value	q-value	pathway	source	external_id	members_input_overlap	members_input_overlap_ge	size	effective_size
1.42703840713e-06	0.0002825536	Laminin interactions	Reactome	R-HSA-30001	LAMC3; LAMA2; HSPG2; LAI	10319; 3339; 284217; 3908	23	23
5.08312407033e-06	0.0005032292	Retinol metabolism - Homo sapiens (human)	KEGG	path:hsa0083	DHRS4L2; CYP2C8; CYP2S1;	1558; 50700; 9249; 317749	68	68
9.69684503611e-06	0.0006399917	Extracellular matrix organization	Reactome	R-HSA-14742	LAMA1; LAMA2; MMP17; C(	10319; 11095; 284217; 390	287	287
1.35159675952e-05	0.0006690403	antigen processing and presentation	BioCarta	mhcpathway	TAP1; HLA-DRA; PSMB9	3122; 6890; 5698	13	13
1.71232525589e-05	0.0006780808	Non-integrin membrane-ECM interactions	Reactome	R-HSA-30001	LAMC3; LAMA2; HSPG2; LAI	10319; 3339; 284217; 3908	42	42
2.71801224752e-05	0.0008969440	Human papillomavirus infection - Homo sapiens	KEGG	path:hsa0516	PRKCI; LAMA1; LAMA2; NOT	10319; 3908; 23545; 24597	331	331
3.44684693206e-05	0.0009581583	Chondroitin sulfate/dermatan sulfate metabolis	Reactome	R-HSA-17931	UST; HSPG2; CSPG5; CHSY1	10090; 3339; 22856; 10675	50	50
3.87134671206e-05	0.0009581583	Degradation of the extracellular matrix	Reactome	R-HSA-14742	COL25A1; MMP17; HSPG2;	11095; 4326; 3339; 84570;	103	103
4.50782454137e-05	0.0009917213	prion pathway	BioCarta	prionpathwa	LAMC3; LAMA2; LAMA1	10319; 284217; 3908	19	19
5.78553845835e-05	0.0011455366	Toxoplasmosis - Homo sapiens (human)	KEGG	path:hsa0514	LAMA1; HLA-DRA; CD40LG;	3122; 284217; 10319; 959;	112	112
6.64605970793e-05	0.0011703768	Viral myocarditis - Homo sapiens (human)	KEGG	path:hsa0541	LAMA1; HLA-DRA; CD40LG;	3122; 284217; 959; 3908	60	59
7.09319322774e-05	0.0011703768	Nervous system development	Reactome	R-HSA-96751	LAMA1; LAMA2; PSPN; EPHA	2044; 3908; 6336; 6710; 86	379	379
0.00016053173860	0.0024450218	Developmental Biology	Reactome	R-HSA-12667	LAMA1; LAMA2; PSPN; EPHA	2044; 3908; 6336; 6710; 86	676	676
0.00018350410835	0.0025952723	Asthma - Homo sapiens (human)	KEGG	path:hsa0531	CCL11; HLA-DRA; CD40LG	3122; 959; 6356	31	30
0.00023667451301	0.0030277966	Phagosome - Homo sapiens (human)	KEGG	path:hsa0414	TAP1; ATP6V0D2; HLA-DRA;	3122; 4153; 6890; 245972;	152	151
0.00024467043453	0.0030277966	Alpha 6 Beta 4 signaling pathway	Wikipathw	WP244	LAMA1; LAMA2; IRS2	284217; 3908; 8660	33	33
0.00029197279328	0.0033757704	RET signaling	Reactome	R-HSA-88536	PSPN; IRS2; GFRA4	8660; 5623; 64096	35	35
0.00031456898270	0.0033757704	ECM-receptor interaction - Homo sapiens (huma	KEGG	path:hsa0451	LAMA1; LAMA2; HSPG2; LAI	284217; 3339; 10319; 3908	88	88
0.00032393757287	0.0033757704	Axon guidance	Reactome	R-HSA-42247	LAMA1; PSPN; EPHA5; SPTB	2044; 6336; 6710; 8660; 28	356	356
0.00040344493695	0.0039941048	O-glycosylation of TSR domain-containing prote	Reactome	R-HSA-51732	ADAMTS20; ADAMTS7; ADA	80070; 11095; 11173	39	39
0.00048066702286	0.0045320033	NOTCH4 Activation and Transmission of Signal to	Reactome	R-HSA-90137	NOTCH4; PSEN2	5664; 4855	9	9
0.00068453142441	0.0055658099	Tyrosine metabolism	EHMN	Tyrosine met	AOC1; CYP2C8; ACAA1; CYP	30; 26; 1558; 29785	108	108
0.00070099499425	0.0055658099	agrin in postsynaptic differentiation	BioCarta	agrpathway	LAMC3; LAMA2; LAMA1	10319; 284217; 3908	47	47
0.00073086393828	0.0055658099	Dermatan sulfate biosynthesis	Reactome	R-HSA-20229	UST; CSPG5	10675; 10090	11	11
0.00073086393828	0.0055658099	CYP2E1 reactions	Reactome	R-HSA-21199	CYP2S1; CYP2C8	29785; 1558	11	11
0.00073086393828	0.0055658099	Alpha6 beta4 integrin-ligand interactions	PID	integrin4_pa	LAMA1; LAMA2	284217; 3908	11	11
0.00087495188031	0.0064163137	POU5F1 (OCT4), SOX2, NANOG activate genes rel	Reactome	R-HSA-28922	SALL1; POU5F1	5460; 6299	12	12
0.00098432375463	0.0069605751	Glycosaminoglycan metabolism	Reactome	R-HSA-16303	CHSY1; UST; HSPG2; CSPG5	10090; 10675; 3339; 22856	119	119
0.00103157659172	0.0070431781	chondroitin sulfate biosynthesis (late stages)	HumanCyc	PWY-6567	UST; CHSY1	10090; 22856	13	13
0.0011705185611	0.0077254225	ECM proteoglycans	Reactome	R-HSA-30001	LAMA1; LAMA2; HSPG2	3339; 284217; 3908	56	56
0.00123232522959	0.0078709804	C21-steroid hormone biosynthesis and metaboli	EHMN	C21-steroid h	CYP2S1; CYP2C8; RDH8	29785; 50700; 1558	57	57
0.0013620308109	0.0084275656	NCAM signaling for neurite out-growth	Reactome	R-HSA-37516	PSPN; SPTB; GFRA4	6710; 5623; 64096	59	59
0.00157575819683	0.0094545491	the visual cycle I (vertebrates)	HumanCyc	PWY-6861	DHRS3; RDH8	50700; 9249	16	16
0.00178161925288	0.0101920939	dermatan sulfate biosynthesis	HumanCyc	PWY-6571	UST; CHSY1	10090; 22856	17	17
0.00180163276272	0.0101920939	IL4-mediated signaling events	PID	il4_2pathway	CCL11; CD40LG; IRS2	8660; 959; 6356	65	65
0.00199956411664	0.0109976026	SREBF and miR33 in cholesterol and lipid homec	Wikipathw	WP2011	ABCA1; NR1H3	10062; 19	18	18
0.0022295036309	0.0119308572	retinol biosynthesis	HumanCyc	PWY-6857	DHRS3; RDH8	9249; 50700	19	19
0.00247134911173	0.0125468493	Glycosaminoglycan biosynthesis - chondroitin s	KEGG	path:hsa005	UST; CHSY1	10090; 22856	20	20
0.00247134911173	0.0125468493	Chondroitin sulfate biosynthesis	Reactome	R-HSA-20228	CHSY1; CSPG5	10675; 22856	20	20
0.00272501234627	0.0131598157	chondroitin sulfate biosynthesis	HumanCyc	PWY-6569	UST; CHSY1	22856; 10090	21	21
0.00272501234627	0.0131598157	Xenobiotics	Reactome	R-HSA-21198	CYP2S1; CYP2C8	29785; 1558	21	21
0.00303330698697	0.0139673205	Notch	INOH	None	NOTCH4; PSEN2; PSMB9	5664; 4855; 5698	78	78
0.00303330698697	0.0139673205	Signaling by Insulin receptor	Reactome	R-HSA-74752	ATP6V0D2; IRS2; ATP6V0A2	245972; 23545; 8660	78	78
0.00412839411135	0.0171911028	Androgen and estrogen biosynthesis and metabc	EHMN	Androgen anc	CYP2S1; CYP2C8; RDH8	50700; 1558; 29785	87	87
0.00416754007883	0.0171911028	A tetrasaccharide linker sequence is required for	Reactome	R-HSA-19714	HSPG2; CSPG5	3339; 10675	26	26
0.00416754007883	0.0171911028	RXR and RAR heterodimerization with other nuc	PID	rxr_vdr_path	ABCA1; NR1H3	10062; 19	26	26
0.00416754007883	0.0171911028	PPAR-alpha pathway	Wikipathw	WP2878	ACAA1; NR1H3	10062; 30	26	26
0.00416754007883	0.0171911028	Insulin receptor recycling	Reactome	R-HSA-77387	ATP6V0D2; ATP6V0A2	245972; 23545	26	26
0.00449028347489	0.0174328652	Signaling by NOTCH4	Reactome	R-HSA-90136	NOTCH4; PSEN2	5664; 4855	27	27
0.00449028347489	0.0174328652	Canonical and non-canonical Notch signaling	Wikipathw	WP3845	NOTCH4; PSEN2	5664; 4855	27	27
0.00449028347489	0.0174328652	Collecting duct acid secretion - Homo sapiens (h	KEGG	path:hsa0496	ATP6V0D2; ATP6V0A2	245972; 23545	27	27
0.00482424014435	0.0177027681	Transcriptional regulation of pluripotent stem c	Reactome	R-HSA-45272	SALL1; POU5F1	5460; 6299	28	28
0.00482802767277	0.0177027681	Rheumatoid arthritis - Homo sapiens (human)	KEGG	path:hsa0532	ATP6V0D2; HLA-DRA; ATP6V	3122; 245972; 23545	93	92
0.00482802767277	0.0177027681	Small cell lung cancer - Homo sapiens (human)	KEGG	path:hsa0522	LAMA1; LAMA2; LAMC3	284217; 10319; 3908	92	92
0.00516932556585	0.0186095720	Interaction between L1 and Ankyrins	Reactome	R-HSA-44509	SPTB; SCN10A	6710; 6336	29	29
0.0054363344807	0.0192213254	Small cell lung cancer	Wikipathw	WP4658	LAMA1; LAMA2; LAMC3	284217; 10319; 3908	96	96
0.00589254683707	0.0198721707	Tight junction interactions	Reactome	R-HSA-42002	PRKCI; PATJ	10207; 5584	31	31
0.00589254683707	0.0198721707	Transferrin endocytosis and recycling	Reactome	R-HSA-91797	ATP6V0D2; ATP6V0A2	245972; 23545	31	31
0.00592150541304	0.0198721707	CD4 T cell receptor signaling-NFkB cascade	INOH	None	HLA-DRA; CARD11; PSMB9	3122; 5698; 84433	99	99
0.00625893157432	0.0198986161	Phase I - Functionalization of compounds	Reactome	R-HSA-21194	AOC1; CYP2C8; CYP2S1	26; 1558; 29785	101	101
0.00625893157432	0.0198986161	Amoebiasis - Homo sapiens (human)	KEGG	path:hsa0514	LAMA1; LAMA2; LAMC3	284217; 10319; 3908	102	101
0.00630043382169	0.0198986161	Nuclear Receptors Meta-Pathway	Wikipathw	WP2882	ACAA1; AMIGO2; POU5F1; I	10062; 5460; 347902; 30; 8	317	317
0.00643187594068	0.0198986161	Leukotriene metabolism	EHMN	Leukotriene r	CYP2S1; CYP2C8; ACAA1	30; 1558; 29785	102	102
0.00643187594068	0.0198986161	L1CAM interactions	Reactome	R-HSA-37376	LAMA1; SPTB; SCN10A	6336; 284217; 6710	102	102
0.0066076573107	0.0201279407	Visual phototransduction	Reactome	R-HSA-21873	HSPG2; DHRS3; RDH8	50700; 3339; 9249	103	103
0.00746886537173	0.0219665150	Nuclear Receptors in Lipid Metabolism and Toxic	Wikipathw	WP299	ABCA1; NR1H3	10062; 19	35	35
0.00746886537173	0.0219665150	ROS and RNS production in phagocytes	Reactome	R-HSA-12225	ATP6V0D2; ATP6V0A2	245972; 23545	35	35
0.00788952315925	0.0219665150	Methadone Action Pathway	SMPDB	SMP00408	CYP2C8; SCN10A	1558; 6336	36	36
0.00832064962308	0.0219665150	Primary immunodeficiency - Homo sapiens (hum	KEGG	path:hsa0534	TAP1; CD40LG	959; 6890	38	37
0.00832064962308	0.0219665150	Allograft rejection - Homo sapiens (human)	KEGG	path:hsa0533	HLA-DRA; CD40LG	3122; 959	38	37
0.00832064962308	0.0219665150	NCAM1 interactions	Reactome	R-HSA-41903	PSPN; GFRA4	5623; 64096	37	37
0.00832064962308	0.0219665150	Type II interferon signaling (IFNG)	Wikipathw	WP619	TAP1; PSMB9	6890; 5698	37	37
0.00832064962308	0.0219665150	NR1H3 & NR1H2 regulate gene expression linked	Reactome	R-HSA-90295	ABCA1; NR1H3	10062; 19	37	37
0.00832064962308	0.0219665150	Vitamin A Deficiency	SMPDB	SMP00336	DHRS3; RDH8	50700; 9249	37	37
0.00832064962308	0.0219665150	Retinol Metabolism	SMPDB	SMP00074	DHRS3; RDH8	50700; 9249	37	37
0.00894237993001	0.0232972529	O-linked glycosylation	Reactome	R-HSA-51731	ADAMTS20; ADAMTS7; ADA	80070; 11173; 11095	115	115

C) A1 Meta-analysis 21 genes								
p-value	q-value	pathway	source	external_i	members_i	members_i	size	effective_size
2.38536645344e-12	2.07526881449e-10	Mycophenolic Acid Metabolism	SMPDB	SMP00652	SLCO1B1; C	10599; 282	16	16
1.33347028529e-09	5.80059574101e-08	Codeine and Morphine Metabo	Wikipathw	WP1604	SLCO1B1; C	1558; 5243	16	16
3.14582251446e-08	6.84216396896e-07	Paclitaxel Action Pathway	SMPDB	SMP00434	ABCB1; SLC	5243; 1244	7	7
3.14582251446e-08	6.84216396896e-07	Docetaxel Action Pathway	SMPDB	SMP00435	ABCB1; SLC	5243; 1244	7	7
6.07443917826e-07	8.80793680848e-06	Drug Induction of Bile Acid Pat	Wikipathw	WP2289	SLCO1B1; A	5243; 1244	17	17
6.07443917826e-07	8.80793680848e-06	Heme degradation	Reactome	R-HSA-189	SLCO1B1; S	10599; 282	17	17
1.56318835575e-06	1.52367031007e-05	Androgen and estrogen biosynt	EHMN	Androgen a	SLCO1B1; C	10599; 154	87	87
1.57621066559e-06	1.52367031007e-05	Irinotecan Action Pathway	SMPDB	SMP00433	SLCO1B1; A	5243; 1059	23	23
1.57621066559e-06	1.52367031007e-05	Irinotecan Metabolism Pathwa	SMPDB	SMP00600	SLCO1B1; A	5243; 1059	23	23
1.79122888299e-06	1.5583691282e-05	Bile secretion - Homo sapiens (I	KEGG	path:hsa04	SLCO1B1; S	28234; 524	90	90
2.95772308163e-06	2.1683949782e-05	Leukotriene metabolism	EHMN	Leukotrien	SLCO1B1; C	10599; 154	102	102
3.02381727866e-06	2.1683949782e-05	Rosiglitazone Metabolism Path	SMPDB	SMP00653	SLCO1B1; C	10599; 155	3	3
3.2401304272e-06	2.1683949782e-05	Metabolism of porphyrins	Reactome	R-HSA-189	SLCO1B1; S	10599; 282	29	29
3.71659309788e-06	2.3095971394e-05	Tyrosine metabolism	EHMN	Tyrosine m	SLCO1B1; C	10599; 154	108	108
4.8261451464e-06	2.79916418491e-05	Pregnane X receptor pathway	Wikipathw	WP2876	SLCO1B1; A	5243; 1244	33	33
1.19132402124e-05	6.4778243655e-05	Nuclear Receptors Meta-Pathw	Wikipathw	WP2882	SLCO1B1; C	2950; 1545	317	317
1.50920890394e-05	7.7235985084e-05	multi-drug resistance factors	BioCarta	mrppathwa	ABCB1; GST	5243; 2950	6	6
2.11163413651e-05	0.000102062316598	Arachidonate Epoxygenase - Ep	Wikipathw	WP678	CYP2C8; GS	1558; 2950	7	7
2.81383544066e-05	0.000128844043862	Synthesis of epoxy (EET) and dih	Reactome	R-HSA-214	CYP2C8; CY	1558; 1545	8	8
3.61563393771e-05	0.000157280076291	Synthesis of (16-20)-hydroxyeic	Reactome	R-HSA-214	CYP2C8; CY	1558; 1545	9	9
4.51685096908e-05	0.000178620924686	Vindesine Action Pathway	SMPDB	SMP00438	ABCB1; ABC	1244; 5243	10	10
4.51685096908e-05	0.000178620924686	Vincristine Action Pathway	SMPDB	SMP00437	ABCB1; ABC	5243; 1244	10	10
5.51730809602e-05	0.000200002418481	Vinblastine Action Pathway	SMPDB	SMP00436	ABCB1; ABC	5243; 1244	11	11
5.51730809602e-05	0.000200002418481	Vinorelbine Action Pathway	SMPDB	SMP00439	ABCB1; ABC	5243; 1244	11	11
6.61682709938e-05	0.000230265583059	Transport of organic anions	Reactome	R-HSA-879	SLCO1B1; S	10599; 282	12	12
7.81522997947e-05	0.000253951944382	Irinotecan pathway	Wikipathw	WP229	SLCO1B1; A	1244; 1059	13	13
7.88126723943e-05	0.000253951944382	Chemical carcinogenesis - Hom	KEGG	path:hsa05	CYP2C8; GS	2950; 1558	83	83
0.000120019651695	0.000360058955084	FOXA2 pathway	Wikipathw	WP5066	SLCO1B1; A	1244; 1059	16	16
0.000120019651695	0.000360058955084	Recycling of bile acids and salts	Reactome	R-HSA-159	SLCO1B1; S	10599; 282	16	16
0.000135941279398	0.000394229710255	Doxorubicin Metabolism Path	SMPDB	SMP00650	ABCB1; ABC	5243; 1244	17	17
0.000152842878718	0.000428946143499	Lamivudine Metabolism Pathw	SMPDB	SMP00649	ABCB1; ABC	1244; 5243	18	18
0.000209409848485	0.000569333025569	Tamoxifen metabolism	Wikipathw	WP691	CYP2C8; CY	1545; 1558	21	21
0.000251988685037	0.000662548948603	Photodynamic therapy-induce	Wikipathw	WP3612	GSTP1; ABC	2950; 1244	23	23
0.000258927175316	0.000662548948603	Metabolism	Reactome	R-HSA-143	CYP2C8; CY	1558; 1244	1954	1952
0.000350065865891	0.000870163723786	Metabolism of lipids	Reactome	R-HSA-556	SLCO1B1; C	1558; 2823	646	645
0.000410873546716	0.000992944404564	Male infertility	Wikipathw	WP4673	ERCC1; ERC	2068; 2067	145	145
0.000431458889616	0.00101451144315	Acetaminophen Metabolism Pa	SMPDB	SMP00640	GSTP1; ABC	2950; 5243	30	30
0.000491376793104	0.00112499423684	Constitutive Androstane Recep	Wikipathw	WP2875	ABCB1; ABC	5243; 1244	32	32
0.000522767157488	0.00116617288978	EPHA forward signaling	PID	epha_fwdp	EPHA6; EPH	285220; 20	33	33
0.000555109483687	0.00120736312702	Porphyrin metabolism	EHMN	Porphyrin	SLCO1B1; S	10599; 282	34	34
0.000588402039424	0.00124856042512	Nuclear Receptors in Lipid Met	Wikipathw	WP299	ABCB1; ABC	5243; 1244	35	35
0.000808015456634	0.00167374630303	Dual Incision in GG-NER	Reactome	R-HSA-569	ERCC1; ERC	2068; 2067	41	41
0.000849516823725	0.00168088460125	Metapathway biotransformatic	Wikipathw	WP702	CYP2C8; GS	1545; 1558	186	186
0.000888743582269	0.00168088460125	Nucleotide Excision Repair	Wikipathw	WP4753	ERCC1; ERC	2068; 2067	43	43
0.000888743582269	0.00168088460125	Formation of Incision Complex	Reactome	R-HSA-569	ERCC1; ERC	2068; 2067	43	43
0.000888743582269	0.00168088460125	Bile acid and bile salt metaboli	Reactome	R-HSA-194	SLCO1B1; S	10599; 282	43	43
0.000930510604558	0.00172243452333	Transport of vitamins, nucleosi	Reactome	R-HSA-425	SLCO1B1; S	10599; 282	44	44
0.000973210651216	0.00176394430533	ABC transporters - Homo sapier	KEGG	path:hsa02	ABCB1; ABC	1244; 5243	45	45
0.00106140298454	0.00188453182969	Nucleotide excision repair - Ho	KEGG	path:hsa03	ERCC1; ERC	2067; 2068	47	47
0.00124890889817	0.00217310148281	Xenobiotics metabolism	EHMN	Xenobiotic	CYP2C8; CY	1558; 1545	51	51
0.00134819529	0.00227881499365	Bile acid biosynthesis	EHMN	Bile acid bi	SLCO1B1; S	10599; 282	53	53
0.00136205034103	0.00227881499365	Biological oxidations	Reactome	R-HSA-211	CYP2C8; GS	1545; 1558	219	219
0.00155776722564	0.00246410452056	Cytochrome P450 - arranged by	Reactome	R-HSA-211	CYP2C8; CY	1558; 1545	57	57
0.00155776722564	0.00246410452056	C21-steroid hormone biosynth	EHMN	C21-steroic	CYP2C8; CY	1558; 1545	57	57
0.00155776722564	0.00246410452056	Proximal tubule transport	Wikipathw	WP4917	ABCB1; ABC	5243; 1244	57	57
0.00161244185773	0.00250504360041	ABC-family proteins mediated t	Reactome	R-HSA-382	ABCB1; ABC	1244; 5243	58	58
0.00172451732262	0.00263215801873	Arachidonic acid metabolism	Reactome	R-HSA-214	CYP2C8; CY	1558; 1545	60	60
0.00184021653685	0.00276032480528	Oxidation by Cytochrome P450	Wikipathw	WP43	CYP2C8; CY	1545; 1558	62	62
0.0020824326762	0.00307070581066	Tryptophan degradation	INOH	None	CYP2C8; CY	1558; 1545	66	66
0.00214523066876	0.0031105844697	Dual incision in TC-NER	Reactome	R-HSA-678	ERCC1; ERC	2068; 2067	67	67
0.00233898353457	0.00328212205657	Global Genome Nucleotide Exc	Reactome	R-HSA-569	ERCC1; ERC	2068; 2067	70	70
0.00233898353457	0.00328212205657	Drug metabolism - cytochrome	KEGG	path:hsa00	CYP2C8; GS	2950; 1558	72	70
0.0024726012722	0.00341454461399	Linoleate metabolism	EHMN	Linoleate m	CYP2C8; CY	1558; 1545	72	72
0.00260976290244	0.0035476464455	EPH-Ephrin signaling	Reactome	R-HSA-268	EPHA6; EPH	285220; 20	74	74
0.00275045523181	0.00368137854104	Metabolism of xenobiotics by c	KEGG	path:hsa00	GSTP1; CYP	2950; 1545	78	76
0.00296808500714	0.00391247569123	Transcription-Coupled Nucleot	Reactome	R-HSA-678	ERCC1; ERC	2068; 2067	79	79
0.00350279146424	0.00454840085655	Transport of small molecules	Reactome	R-HSA-382	SLCO1B1; S	10599; 282	641	641
0.00443759954846	0.00567751706935	Nucleotide Excision Repair	Reactome	R-HSA-569	ERCC1; ERC	2068; 2067	97	97
0.00452735652966	0.00570840605913	Arachidonic acid metabolism	EHMN	Arachidoni	CYP2C8; CY	1545; 1558	98	98
0.00480168347788	0.00596780660822	Phase I - Functionalization of cc	Reactome	R-HSA-211	CYP2C8; CY	1545; 1558	101	101
0.00660471006997	0.00809309543785	DNA Repair Pathways Full Netw	Wikipathw	WP4946	ERCC1; ERC	2068; 2067	119	119
0.00715255105096	0.00864266585324	Metabolism of steroids	Reactome	R-HSA-895	SLCO1B1; S	10599; 282	124	124
0.00941576185678	0.0112215244047	NRF2 pathway	Wikipathw	WP2884	GSTP1; ABC	1244; 2950	143	143

Supplementary Figure S1.

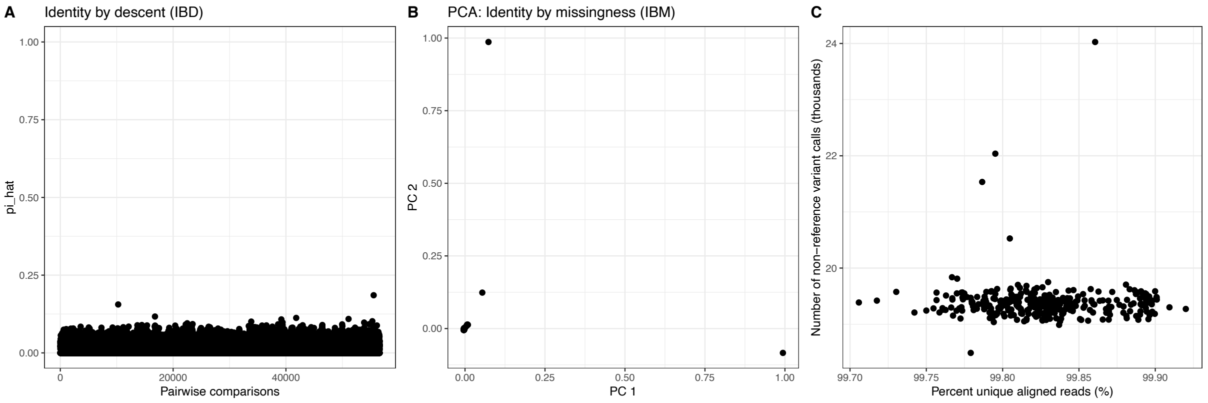

After removal of five patients and variant filtration in PLINK on the remaining 337 patients, the quality metrics identity by descent (IBD), identity by missingness (IBM), as well as the number of non-reference variants versus the percent of unique aligned reads were investigated. A) Shows IBD, or how variants are shared between samples, and in this case, there was no identifiable contamination or evident sample-to-sample relationships. B) Shows IBM, which uses the samples of missing genotypes for clustering, and only three samples deviated from the remaining tightly clustered samples in the original. C) Shows the number of non-reference variants on the y-axis, with an average of 19,383, and three samples with about 2000–4000 more non-reference variants than the others, and the percent of unique aligned reads on the x-axis (which was very similar from sample to sample; all of which were within 99.70–99.95%.) The three samples outside of the IBM cluster were also the ones with the most non-reference variants, which explains their deviation, and they were therefore still deemed reliable and included in the study.

Supplementary Figure S2.

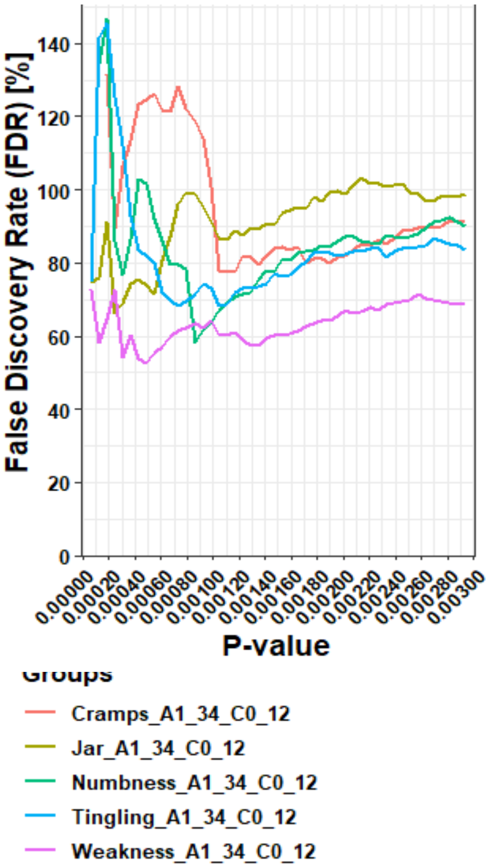

Permutation and false discovery rate for the neuropathy symptoms Visualizes the permutations and the false discovery rate (FDR) to guide the choice of p-value thresholds to select which SNVs merit further evaluation. This is based on the results from permutations (n=1000) using randomly-shuffled values of the five TIPN symptoms/phenotypes and plots the estimated FDR when comparing the permutations to the true test at different p-value levels ranging from 0.005–0.00001. For the five phenotypes, the FDR decreases with a lower p-value threshold until p-values reach ≤0.0012–0.0005, where the FDRs quickly starts to fluctuate and increase creating a minimum of FDR. This minimum of FDR was found (0.000875, 0.001125, 0.001125, 0.0005, and 0.0005) for respectively *numbness of feet* (green), *tingling of feet* (blue), *cramps in feet* (red), *difficulty opening a jar* (brown), and *difficulty climbing stairs because of weakness in legs* (magenta). These thresholds for *numbness of feet*, *tingling of feet*, *cramps in feet*, *difficulty opening a jar*, and *difficulty climbing stairs because of weakness in legs* will still include false positives, but the important true positives will be kept intact using this approach.

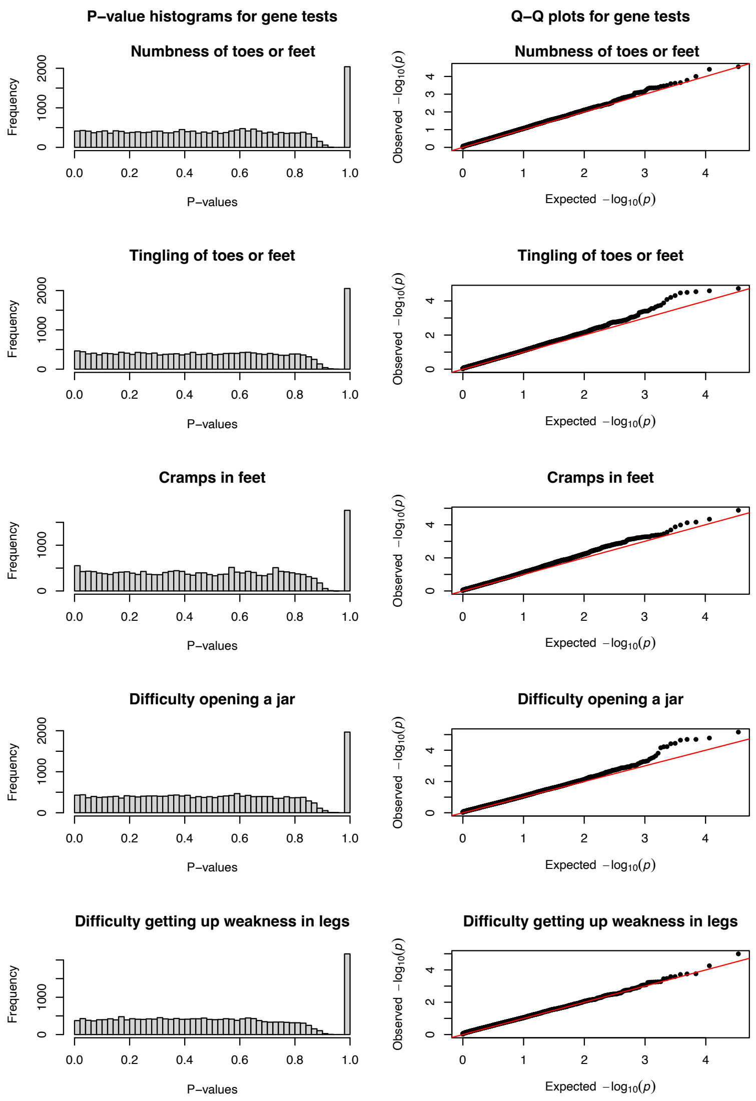

Supplementary figure S3. Visualizes the p-values of the gene tests for *numbness of feet, tingling of feet, cramps in feet, difficulty opening a jar, difficulty climbing stairs* using histograms (to the left) and Q-Q plots (to the right).

Supplementary Figure S4:

A. CV Error Table

K	CV ERROR
1	0.15025
2	0.15365
3	0.15675
4	0.15958
5	0.16211

B. ADMIXTURE plot

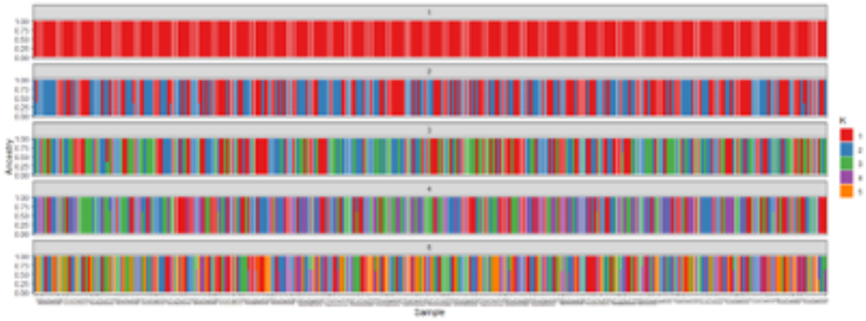

Cross-validation Error Table and ADMIXTURE plot for population stratification analysis Visualizes the results from the ADMIXTURE analysis using the K= 1-5. A) Table with the cross-Validation (CV) error for each K=1-5 indicating which K to use in further analysis. B) ADMIXTURE plot of the grouping of all 337 samples for each K=1-5. Each bar represents one patient, and the color of the bar is the estimated ancestry fractions for each group. K increases from 1 to 5 from top to bottom of the combined plots.
