# Supplementary material for: Prediction models of persistent taxane-induced peripheral neuropathy among breast cancer survivors using whole-exome sequencing
Source: NPJ Precis Oncol. 2024 May 16;8:102. doi: 10.1038/s41698-024-00594-x (PMC11099113; doi:10.1038/s41698-024-00594-x)
Supplement: Supplementary file 2 — REPORTING SUMMARY [file 41698_2024_594_MOESM2_ESM.pdf]

nature portfolio

Corresponding author(s): Dr Kristina Engvall

Last updated by author(s): Nov 10, 2023

Reporting Summary

Nature Portfolio wishes to improve the reproducibility of the work that we publish. This form provides structure for consistency and transparency in reporting. For further information on Nature Portfolio policies, see our Editorial Policies and the Editorial Policy Checklist.

Statistics

For all statistical analyses, confirm that the following items are present in the figure legend, table legend, main text, or Methods section.

n/a	Confirmed	
☐	☒	The exact sample size (n) for each experimental group/condition, given as a discrete number and unit of measurement
☐	☒	A statement on whether measurements were taken from distinct samples or whether the same sample was measured repeatedly
☐	☒	The statistical test(s) used AND whether they are one- or two-sided<br>*Only common tests should be described solely by name; describe more complex techniques in the Methods section.*
☐	☒	A description of all covariates tested
☐	☒	A description of any assumptions or corrections, such as tests of normality and adjustment for multiple comparisons
☐	☒	A full description of the statistical parameters including central tendency (e.g. means) or other basic estimates (e.g. regression coefficient) AND variation (e.g. standard deviation) or associated estimates of uncertainty (e.g. confidence intervals)
☐	☒	For null hypothesis testing, the test statistic (e.g. F, t, r) with confidence intervals, effect sizes, degrees of freedom and P value noted<br>*Give P values as exact values whenever suitable.*
☒	☐	For Bayesian analysis, information on the choice of priors and Markov chain Monte Carlo settings
☒	☐	For hierarchical and complex designs, identification of the appropriate level for tests and full reporting of outcomes
☒	☐	Estimates of effect sizes (e.g. Cohen's d, Pearson's r), indicating how they were calculated

Our web collection on statistics for biologists contains articles on many of the points above.

Software and code

Policy information about availability of computer code

Data collection	Not applicable
Data analysis	For building and validation of prediction models, R version 4.0.3 43 was used. All models were built through logistic regression with binominal distribution using the function glm in the R package stats (version 3.6.2).

For manuscripts utilizing custom algorithms or software that are central to the research but not yet described in published literature, software must be made available to editors and reviewers. We strongly encourage code deposition in a community repository (e.g. GitHub). See the Nature Portfolio guidelines for submitting code & software for further information.

Data

Policy information about availability of data

All manuscripts must include a data availability statement. This statement should provide the following information, where applicable:

- Accession codes, unique identifiers, or web links for publicly available datasets
- A description of any restrictions on data availability
- For clinical datasets or third party data, please ensure that the statement adheres to our policy

The data generated in this study are available upon reasonable request from the corresponding author.

Research involving human participants, their data, or biological material

Policy information about studies with human participants or human data. See also policy information about sex, gender (identity/presentation), and sexual orientation and race, ethnicity and racism.

Reporting on sex and gender	Only female breast breast cancer survivors.
Reporting on race, ethnicity, or other socially relevant groupings	Not applicable or reported.
Population characteristics	Breast cancer survivors in median 3.6 years after treatment. All received taxane chemotherapy. Median age 62 years. Diabetes mellitus and BMI were also included as covariates.
Recruitment	Postal questionnaire for fenotype data and the following postal information about the performed sub-study. Possibility to receive oral information from and possibility to ask questions to study nurse or doctor before leaving written informed consent to participate.
Ethics oversight	Regional board of Ethics, Linköping University

Note that full information on the approval of the study protocol must also be provided in the manuscript.

Field-specific reporting

Please select the one below that is the best fit for your research. If you are not sure, read the appropriate sections before making your selection.

☒ Life sciences ☐ Behavioural & social sciences ☐ Ecological, evolutionary & environmental sciences

For a reference copy of the document with all sections, see nature.com/documents/nr-reporting-summary-flat.pdf

Life sciences study design

All studies must disclose on these points even when the disclosure is negative.

Sample size	N=337 in this study. Sample size was determined for the main study (Engvall BJC 2021).
Data exclusions	N=362 provided a blood sample. 23 were excluded due to more advanced stage of disease. 2 excluded due to missing data on neuropathy questions.
Replication	We used a training cohort and a separate test cohort, the individuals in the cohorts were not mixed. The separate test cohort was used for validation.
Randomization	The training cohort and test cohort were randomised aiming at as even groups as possible based on the covariates.
Blinding	Not relevant.

Reporting for specific materials, systems and methods

We require information from authors about some types of materials, experimental systems and methods used in many studies. Here, indicate whether each material, system or method listed is relevant to your study. If you are not sure if a list item applies to your research, read the appropriate section before selecting a response.

Materials & experimental systems

n/a	Involved in the study	
☒	☐	Antibodies
☒	☐	Eukaryotic cell lines
☒	☐	Palaeontology and archaeology
☒	☐	Animals and other organisms
☐	☒	Clinical data
☒	☐	Dual use research of concern
☒	☐	Plants

Methods

n/a	Involved in the study	
☒	☐	ChIP-seq
☒	☐	Flow cytometry
☒	☐	MRI-based neuroimaging

Clinical data

Policy information about clinical studies

All manuscripts should comply with the ICMJE guidelines for publication of clinical research and a completed CONSORT checklist must be included with all submissions.

Clinical trial registration	Not an interventional study.
Study protocol	A full desciption of the main study has previously been published: Engvall, K., Gréen, H., Fredriksson, M. & Åvall-Lundqvist, E. Persistent neuropathy among early-stage breast cancer survivors in a population-based cohort. British journal of cancer (2021).
Data collection	Fenotype based on EORTC CIPN20 in a questionnaire study in 2017. Genotype from blood samples collected in 2018.
Outcomes	We leveraged logistic regression models to develop and validate polygenic prediction models to estimate the risk of persistent peripheral neuropathy symptoms in a training cohort and validation cohort taking clinical risk factors into account.

Plants

Seed stocks	N/A
Novel plant genotypes	*Describe the methods by which all novel plant genotypes were produced. This includes those generated by transgenic approaches, gene editing, chemical/radiation-based mutagenesis and hybridization. For transgenic lines, describe the transformation method, the number of independent lines analyzed and the generation upon which experiments were performed. For gene-edited lines, describe the editor used, the endogenous sequence targeted for editing, the targeting guide RNA sequence (if applicable) and how the editor was applied.*
Authentication	*Describe any authentication procedures for each seed stock used or novel genotype generated. Describe any experiments used to assess the effect of a mutation and, where applicable, how potential secondary effects (e.g. second site T-DNA insertions, mosiacism, off-target gene editing) were examined.*
